# Machine learning reveals prominent spontaneous behavioral changes and treatment efficacy in humanized and transgenic Alzheimer’s disease models

**DOI:** 10.1016/j.celrep.2024.114870

**Published:** 2024-10-19

**Authors:** Stephanie R. Miller, Kevin Luxem, Kelli Lauderdale, Pranav Nambiar, Patrick S. Honma, Katie K. Ly, Shreya Bangera, Mary Bullock, Jia Shin, Nick Kaliss, Yuechen Qiu, Catherine Cai, Kevin Shen, K. Dakota Mallen, Zhaoqi Yan, Andrew S. Mendiola, Takashi Saito, Takaomi C. Saido, Alexander R. Pico, Reuben Thomas, Erik D. Roberson, Katerina Akassoglou, Pavol Bauer, Stefan Remy, Jorge J. Palop

**Affiliations:** 1 Gladstone Institute of Neurological Disease, San Francisco, CA 94158, USA; 2 Department of Neurology, Weill Institute for Neurosciences, University of California, San Francisco, San Francisco, CA 94158, USA; 3 German Center for Neurodegenerative Diseases (DZNE), 39118 Bonn and Magdeburg, Germany; 4 Department of Cellular Neuroscience, Leibniz Institute for Neurobiology, 39118 Magdeburg, Germany; 5 Center for Behavioral Brain Sciences (CBBS), 39106 Magdeburg, Germany; 6 Center for Neurodegeneration and Experimental Therapeutics, Alzheimer’s Disease Center, Department of Neurology, University of Alabama at Birmingham, Birmingham, AL 35233, USA; 7 Center for Neurovascular Brain Immunology at Gladstone and UCSF, San Francisco, CA 94158, USA; 8 Department of Neurocognitive Science, Nagoya City University Graduate School of Medical Sciences, Nagoya 467-8601, Japan; 9 Laboratory for Proteolytic Neuroscience, RIKEN Center for Brain Science, Wako-shi 351-0198, Japan; 10 Gladstone Institute of Data Science and Biotechnology, San Francisco, CA 94158, USA; 11 German Center for Mental Health (DZPG), 39118 Magdeburg, Germany; 12 Lead contact

## Abstract

Computer-vision and machine-learning (ML) approaches are being developed to provide scalable, unbiased, and sensitive methods to assess mouse behavior. Here, we used the ML-based variational animal motion embedding (VAME) segmentation platform to assess spontaneous behavior in humanized *App* knockin and transgenic APP models of Alzheimer’s disease (AD) and to test the role of AD-related neuroinflammation in these behavioral manifestations. We found marked alterations in spontaneous behavior in *App*^NL-G-F^ and 5xFAD mice, including age-dependent changes in motif utilization, disorganized behavioral sequences, increased transitions, and randomness. Notably, blocking fibrinogen-microglia interactions in 5xFAD-*Fgg*^γ390–396A^ mice largely prevented spontaneous behavioral alterations, indicating a key role for neuroinflammation. Thus, AD-related spontaneous behavioral alterations are prominent in knockin and transgenic models and sensitive to therapeutic interventions. VAME outcomes had higher specificity and sensitivity than conventional behavioral outcomes. We conclude that spontaneous behavior effectively captures age- and sex-dependent disease manifestations and treatment efficacy in AD models.

## In brief

Miller et al. use the VAME machine-learning platform to reveal robust spontaneous behavioral deficits in Alzheimer’s disease mouse models. These deficits were ameliorated by a neuroinflammation-related therapeutic intervention and provided higher specificity and sensitivity than conventional approaches. VAME outcomes captured early disease manifestations and treatment efficacy in Alzheimer’s models.

## Graphical Abstract

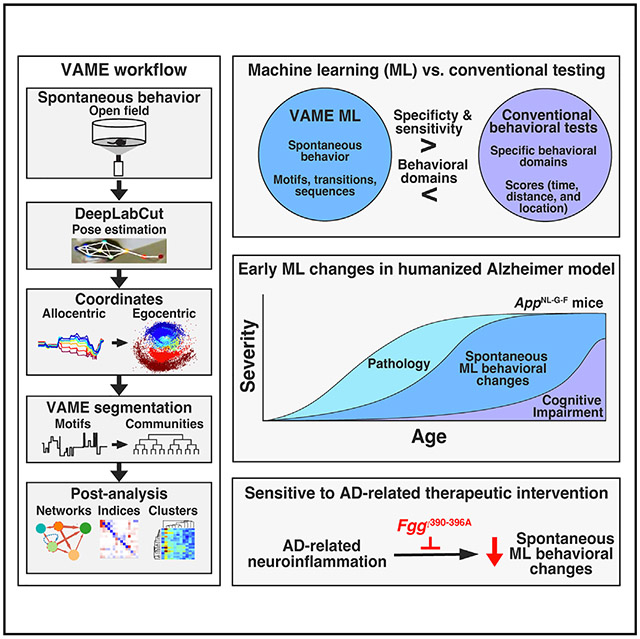

## INTRODUCTION

Behavioral alterations, the defining manifestations of neurological disorders, are intricate and multifaceted, posing challenges to accurate definition and measurement. Thus, our knowledge of disease-induced behavioral alterations is incomplete and largely limited to domain-specific and task-oriented behavioral tests. Analyzing complete sequences of spontaneous behavior can offer deep insight into disease-induced behavioral changes and may provide unbiased and scalable measures of brain dysfunction to evaluate disease progression and therapeutic interventions.^1^ Until recently, this has not been feasible. However, advances in computer-vision and machine-learning (ML) techniques for pose estimation (DeepLabCut,^2,3^ SLEAP,^4^ and LightningPose^5^) and behavioral segmentation (MoSeq^6^ and variational animal motion embedding [VAME]^7,8^) make it possible to deconstruct full sequences of spontaneous mouse behavior into brief postural units (motifs or syllables) and reveal their sequence and hierarchical structure. These emerging methods expand the depth, breadth, and sensitivity of behavioral quantifications and may provide insights into disease pathogenesis.^9^

Alzheimer’s disease (AD) begins several decades before the clinical onset of dementia with progressive amyloid accumulation preceding significant tau pathology and neurodegeneration.^10,11^ During this extended preclinical period prior to the dementia diagnosis, many patients develop behavioral or neuropsychiatric changes, including agitation, irritability, decreased motivation, loss of empathy, and depression, which increase the risk of transition to mild cognitive impairment and AD.^12-14^ Thus, these behavioral changes may be the earliest symptoms of preclinical stages of AD.^13,14^ This extended period of incipient and subtle behavioral alterations holds significant promise for investigations of early AD pathogenesis and therapeutic interventions, particularly given the recent development of ML approaches that may effectively harness both large-scale and precise behavioral data.

Humanized *App* knockin (KI) and APP transgenic mouse models mimic certain aspects of AD pathogenesis, including progressive amyloidosis, gliosis, and neuroinflammation, as well as network hyperexcitability and cognitive impairment, albeit to different degrees of severity across models and paradigms.^15-27^ However, our understanding of alterations in spontaneous behavior in AD models has been limited by the technical challenges of assessing non-task-oriented behavior. Previously, we described the VAME ML architecture and identified motif usage alterations in APP/PS1 transgenic mice.^7^ Here, we advanced the development of our VAME ML pipeline with analytical tools to assess organization of behavioral sequences of spontaneous behavior, disease progression, and sex-dependent effects in humanized *App*^NL-G-F^ KI and 5xFAD transgenic mice. We also validated spontaneous behavioral outcomes for assessing therapeutic interventions by testing the role of AD-related neuroinflammation in 5xFAD mice with or without *Fgg*^γ390–396A^ expression, which inhibits fibrinogen-microglia interactions and reduces AD-related neuroinflammation.^21,22,28^ We conclude that spontaneous behavior effectively captures age- and sex-dependent early disease manifestations and treatment efficacy in AD models with higher sensitivity and specificity than conventional behavioral outcomes.

## RESULTS

### Aged *App*^NL-G-F^ mice have mild spatial memory deficits and severe amyloidosis and gliosis

**Figure 1.**
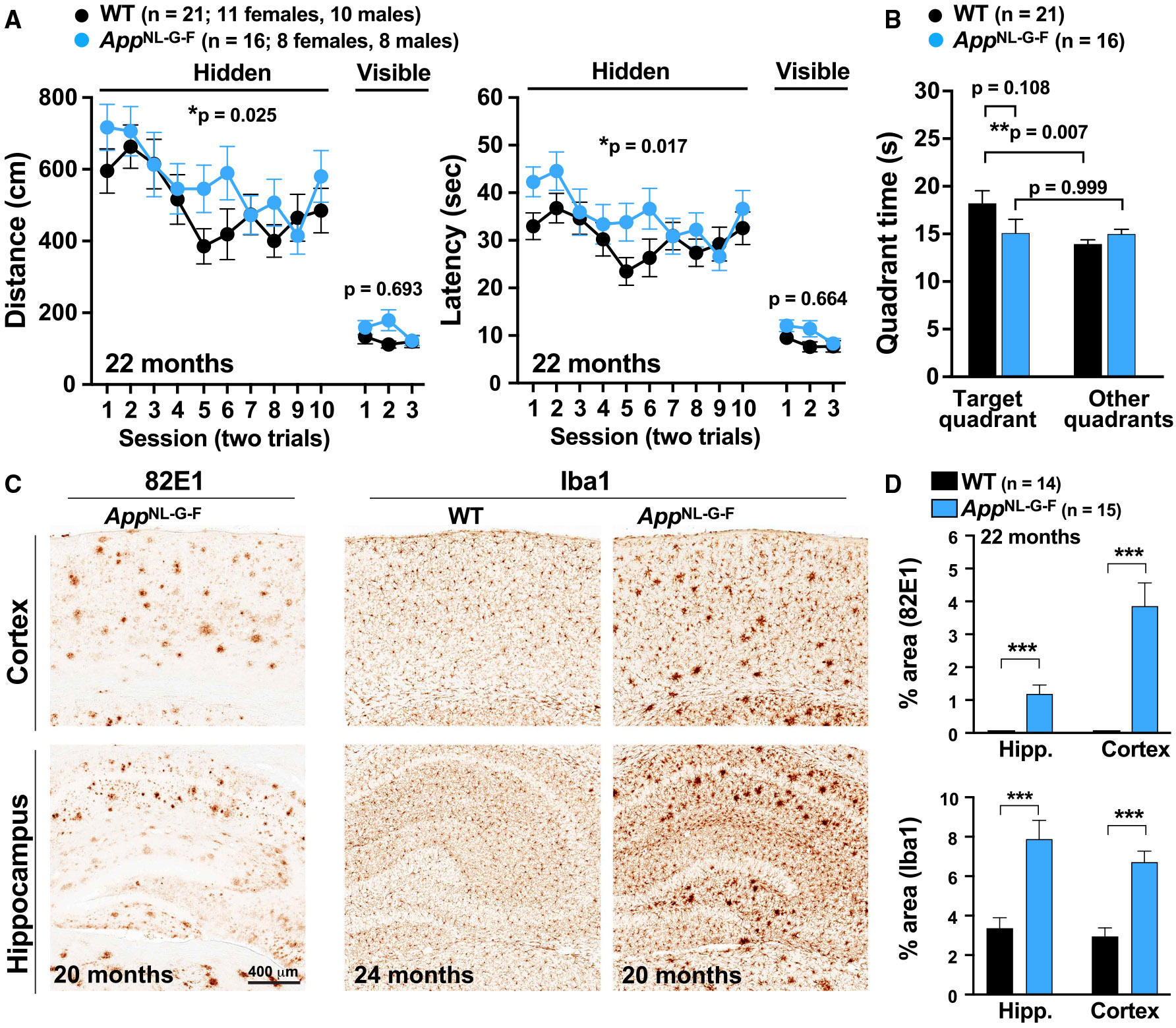
Aged *App*^NL-G-F^ mice show robust AD-related pathology and mild impairments in the Morris water maze Spatial learning and memory of 22-month-old *App*^NL-G-F^ mice (*n* = 16; eight females and eight males) and WT littermate controls (*n* = 21; 11 females and 10 males) were tested in the Morris water maze. (A) Distance swum (left) and latency (right) in the hidden (spatial) and visible (cued) platform components of the Morris water maze test. *App*^NL-G-F^ mice had mild deficits in distances (*p* = 0.025) and latencies (*p* = 0.017) in the spatial component of the Morris water maze. *p* values were determined by repeated two-way ANOVA. (B) Time spent in the target (platform removed) and nontarget quadrants during the 24-h probe trial after 10 sessions of hidden training. *App*^NL-G-F^ and WT mice did not perform differently at the target quadrant (*p* = 0.108). However, WT (*p* = 0.007), but not *App*^NL-G-F^ (*p* = 0.999), mice had a target quadrant preference. *p* values were determined by one-way ANOVA and Bonferroni *post hoc* test. (C) Representative images of hippocampus and cortex stained for 82E1-positve Aβ deposits and Iba1-positive microglia show severe amyloidosis and microgliosis in 22-month-old *App*^NL-G-F^ mice. (D) Quantification of hippocampal and cortical area (%) occupied by 82E1-positve Aβ deposits or by Iba1-positive microglia in 22-month-old *App*^NL-G-F^ (*n* = 14) and WT (*n* = 15) mice. ***p < 0.01 by Student’s t test. Values are mean ± SEM.

First, we used the Morris water maze test to determine whether aged *App*^NL-G-F^ mice develop spatial learning and memory deficits. Consistent with previous findings,^16-19,23^ 22-month-old *App*^NL-G-F^ mice exhibited mild learning and memory deficits in the Morris water maze relative to their wild-type (WT) littermate controls (Figures 1A and 1B). In the hidden platform (spatial) component, *App*^NL-G-F^ mice showed deficits in distance (*p* = 0.025) and latency (*p* = 0.017) (Figure 1A). In the probe trial (platform removed), *App*^NL-G-F^ mice had no preference for the target quadrant (Figure 1B), indicating memory impairment. However, their time in the target quadrant was not significantly different from WT littermate controls (*p* = 0.108, Figure 1B). As expected,^15^ 22-month-old *App*^NL-G-F^ mice had robust AD-related pathology, including amyloid deposition and neuroinflammation (Figures 1C and 1D). Unlike APP transgenic mice,^21,25^ *App* KI mice seem to develop milder cognitive changes.^16-19,23^ As suggested,^15,20^ *App* KI mice may model pre-clinical stages of AD, characterized by progressive amyloidosis and gliosis without prominent cognitive deterioration, tau pathology, or neurodegeneration.

### VAME deconstructs sequences of mouse behavior into postural motifs

**Figure 2.**
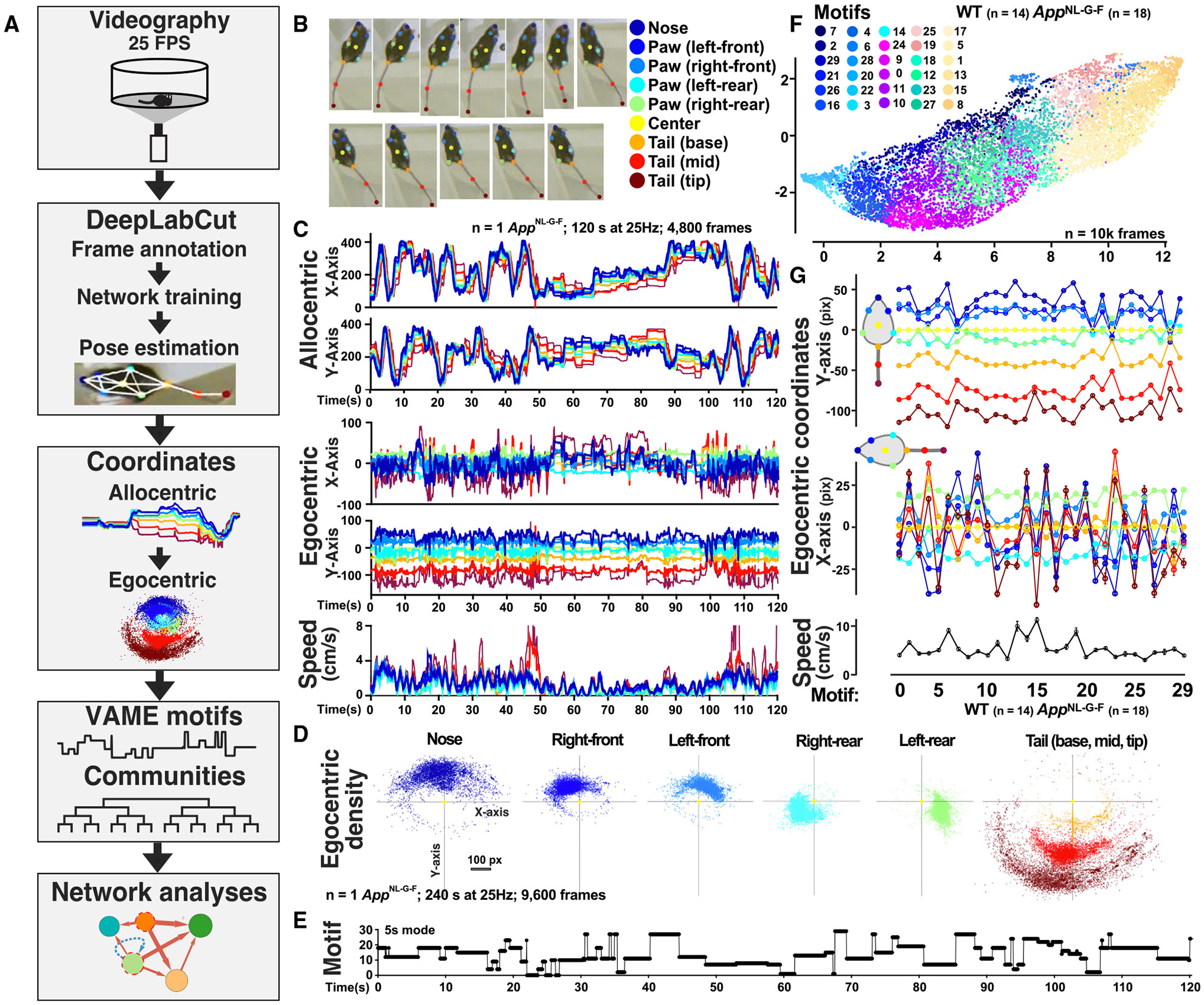
VAME segmentation of behavioral motifs during exploration in an open arena (A) Workflow. Mice were recorded from below at 25 frames per second (25 Hz) for 25 min (~37,500 frames/mouse) in a circular arena 12 inches in diameter. DeepLabCut (DLC) was used for pose estimation of nine body parts. VAME was used to identify behavioral motifs. Custom scripts were used to define egocentric coordinates, communities, and transition sequences. (B) DLC pose estimation of nine defined body parts (ventral view). (C) Allocentric (relative to arena) and egocentric (relative to mouse center) x and y coordinates (pixels) and allocentric speeds of the nine defined body parts (B) of a representative *App*^NL-G-F^ mouse recorded over 2 min at 25 Hz. (D) Egocentric coordinates relative to center (yellow) for the defined body parts (B) over 4 min in a representative *App*^NL-G-F^ mouse (1 point per 40 ms; 9,600 frames). Note the left-right and rostral-caudal motion range of all body parts. (E) Motif sequence (5-s mode) for (C). VAME model trained on egocentric coordinates for nose, paws, center, and tail base. (F) UMAP representation of the 30 motifs identified by VAME for all mice (*n* = 32; 18 *App*^NL-G-F^ and 14 WT mice). Motifs occupy distinct locations with smooth transitions as expected for continuous behavioral sequences; 10,000 random frames shown from the entire cohort and duration. (G) Egocentric coordinates in the rostral-caudal (y axis, top) and left-right (x axis, middle) axes of the nine body parts for the 30 identified motifs (*n* = frames; 32 mice). Points are the average location across all mice for the first 20 frames (800 ms) from motif identification. Allocentric center speed (bottom) across the full cohort (*n* = frames; 32 mice). Motifs show specific and distinct coordinates and speeds. Values are mean ± SEM.

To study spontaneous behavioral manifestations in *App*^NL-G-F^ mice, we built upon our ML pipeline and deconstructed complete sequences of spontaneous mouse behavior during exploration of an open arena. To improve upon the original VAME method,^7^ we used refined algorithms for egocentric alignment of body parts, clustering of motifs into behavioral communities, and advanced kinematic and network analyses (see STAR Methods). Using these revised approaches, we studied spontaneous behavior for 25 min in an open arena to capture innate spontaneous behaviors that are not prompted by an external behavioral task or reward. Ventral-view videos (25 frames per second) of 13-month-old *App*^NL-G-F^ mice (*n* = 18) and littermate WT controls (*n* = 14) (Figure 2A) allowed continuous visibility of limbs and other body parts that define mouse behavior. A DeepLabCut supervised neural network was used for pose estimation of nine body parts: nose, four paws, center, and the base, mid-point, and tip of the tail (Figure 2B). We then generated allocentric (relative to arena) and egocentric (relative to mouse center) x and y coordinates and speeds of the defined body parts (Figure 2C).

Egocentric x and y coordinates captured full motion of the nine body parts within the mouse left-right and rostral-caudal axes, respectively, relative to the mouse center (Figure 2D). Egocentric coordinates of seven body parts (nose, four paws, center, and tail base) were fed into VAME to train an unsupervised dynamical model, which assessed postural patterns across a sliding window of 16 frames (640-ms window) to identify 30 distinct postural motifs.^7^ We compared *k*-means and hidden Markov model (HMM) algorithms for clustering the latent space embeddings into motifs and performed a logistic regression (classifier) analysis to assess the probability of either approach to correctly classify *App*^NL-G-F^ and WT mice (Figure S1A). The *k*-mean algorithm had a higher overall probability of classifying genotype correctly than HMM (93.8% vs. 90.6%) and was used to generate a unique sequence of motifs for each mouse (Figure 2E). Thus, each motif represents a brief sequence of behavior that is consistently identified across mice and over time.

To assess motif specificity across sex and genotypes, we used uniform manifold approximation and projection (UMAP) and kinematic analyses. We found that each motif had a well-delimited UMAP location with smooth transitions between motifs (Figure 2F), indicating specificity of motif features and continuity of behavioral postural motion across motifs. Notably, motifs of each mouse fully overlapped with the corresponding motifs of the other mice regardless of sex and genotype (Figure S1B), indicating that motif identification was consistent throughout the cohort. Kinematics analyses of egocentric coordinates of the 30 motifs also revealed high specificity regardless of sex and genotype (Figures 2G, S1C, and S2), enabling us to precisely study mouse behavior. Thus, VAME can identify postural patterns in motion time series without supervised annotation, permitting reliable and unbiased behavioral segmentation.

### Male and female *App*^NL-G-F^ mice develop prominent age-dependent alterations in spontaneous behavior

**Figure 3.**
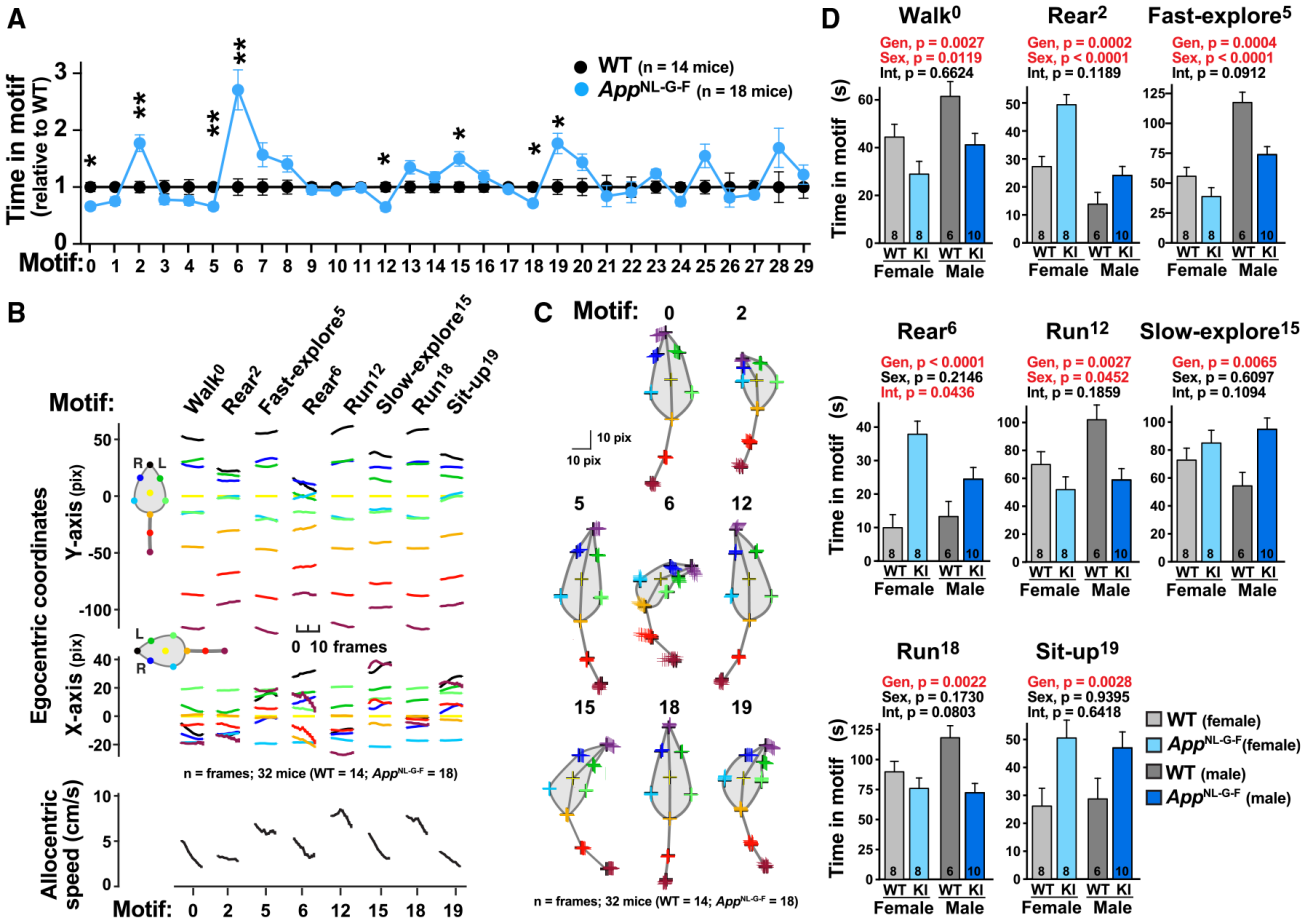
Middle-age *App*^NL-G-F^ mice display robust behavioral alterations in motif use Spontaneous behavior of 13-month-old *App*^NL-G-F^ mice (*n* = 18; eight females and 10 males) and WT littermate controls (*n* = 14; eight females and six males) was recorded for 25 min in a circular open arena. (A) Thirty behavioral motifs were identified by VAME. Motif use (relative to sex-matched WT littermates) of 30 identified motifs for all mice. Relative to littermate controls, *App*^NL-G-F^ mice displayed usage alterations in eight motifs. **q < 0.01, *q < 0.05 by false discovery rate with Benjamini-Hochberg correction for multiple comparisons (FDR-BH). (B) Kinematic analyses of egocentric coordinates of the defined body parts for the first 10 frames (400 ms) of the eight motifs significantly affected in *App*^NL-G-F^ mice (A, *q* < 0.05 by FDR-BH; *n* = frames; 32 mice). (C) Schematic of body part positions for the indicated motifs. Data points are the average location for 10 frames after motif identification (*n* = frames; 32 mice). Motifs show precise and distinct coordinates. (D) Absolute motif use (in seconds) by genotype and sex for the eight motifs altered in *App*^NL-G-F^ mice (A). Males and females differed in their use of motifs 0, 2, 5, and 12, but *App*^NL-G-F^ expression affected both sexes similarly. *p* values were determined by two-way (genotype and sex) ANOVA. Values are mean ± SEM.

Next, we determined whether VAME can detect spontaneous behavior deficits in young (6 months) and middle-aged (13 months) *App*^NL-G-F^ mice compared to age-matched littermate controls. While 6-month-old *App*^NL-G-F^ mice did not display deficits (Figure S1D), we found that motif usage was markedly altered in 13-month-old *App*^NL-G-F^ mice, with significant (*q* < 0.05) alterations in eight of 30 identified motifs, as shown by false discovery rate test with Benjamini-Hochberg correction for multiple comparisons (FDR-BH) (Figure 3A). Kinematic and video analyses of the eight motifs affected in *App*^NL-G-F^ mice revealed that the motifs belong to the following generic behavioral categories: walk (motif 0), fast explore (motif 5), slow explore (motif 15), rear (motifs 2, 6), run (motifs 12 and 18), and sit up (motif 19) (Figures 3B, 3C, S2, and S3A). Thus, *App*^NL-G-F^ mice exhibit robust age-dependent behavioral alterations in spontaneous behavior.

To assess gene and sex interactions, we analyzed the time spent in the eight *App*-affected motifs by two-way ANOVA (sex and genotype). Notably, VAME identified consistent and robust differences in motif use between male and female controls (Figure 3D). However, the effects of *App* genotype and the direction of disease-associated changes in motif use were remarkably consistent across females and males, indicating that VAME can resolve sex and genotype interactions.

### Hierarchical clustering of motifs reveals behavioral communities

**Figure 4.**
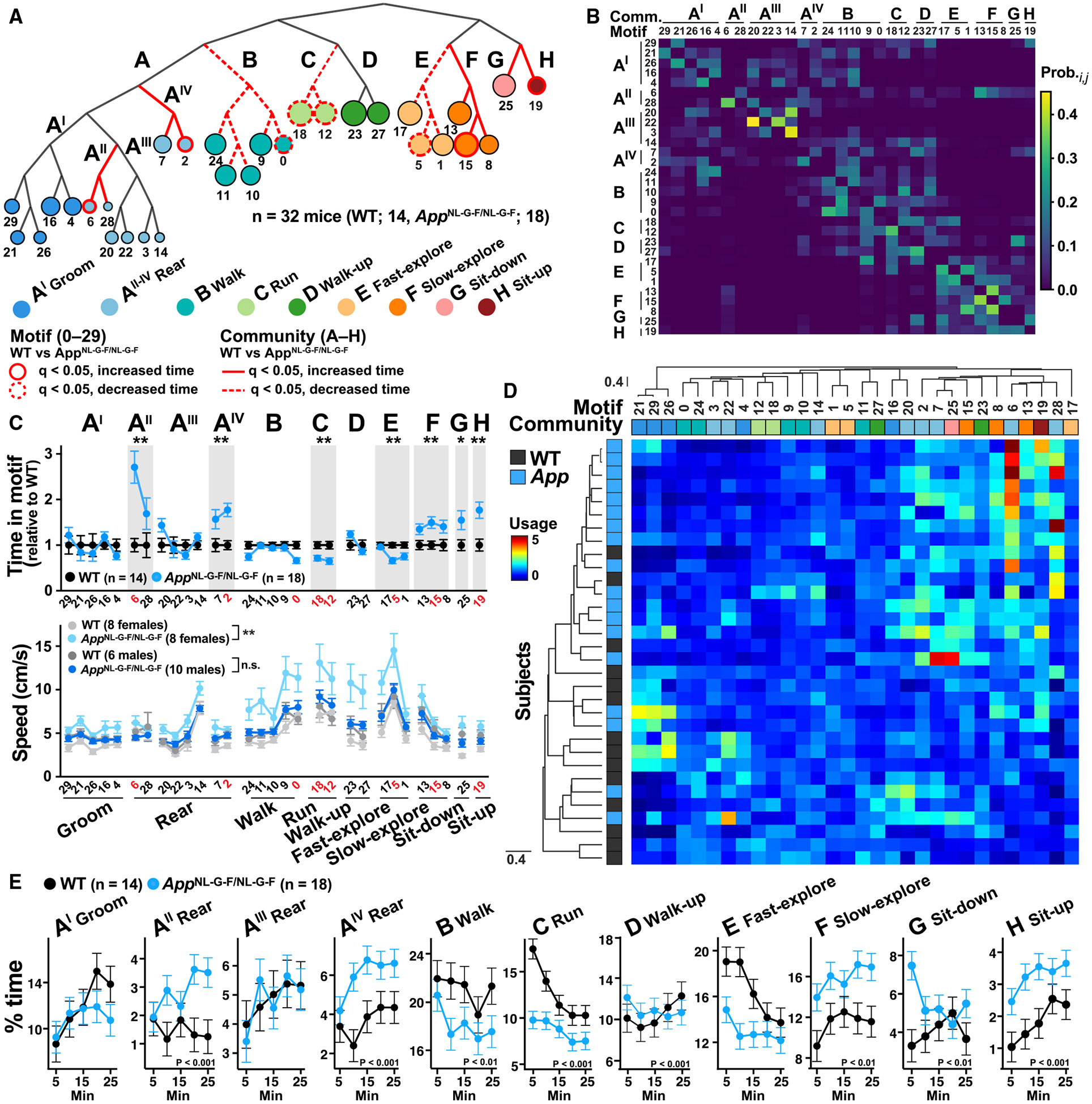
Hierarchical clustering of motifs identifies behavioral communities and reveals experience-dependent alterations in *App*^NL-G-F^ mice (A) Cost-function-based hierarchical organization of motifs into 11 communities based on motif transitions and motif use at the cohort level (*n* = 32; 18 *App*^NL-G-F^ and 14 WT mice). Motifs with higher probability of transitions and closer hierarchical distance were clustered into communities on the dendrogram. Node size is proportional to motif use. Red borders and branches indicate significant differences between *App*^NL-G-F^ and WT mice by FDR-BH for 30 motifs and 11 communities, respectively. (B) Matrix depicting the probability of forward transitions from ${\mathrm{motif}}_{i}$ (rows) to ${\mathrm{motif}}_{j}$ (columns) at the cohort level (*n* = 32; 18 *App*^NL-G-F^ and 14 WT mice). Motifs were organized according to the dendrogram order found in (A). (C) Motif use (relative to sex-matched WT littermates) (top) and speeds (bottom) organized by community. Top: motifs in the same community had reliably similar *App*^NL-G-F^ effects, consistent with the notion that each community reflects a tightly associated set of postural units. **q < 0.01, *q < 0.05 by FDR-BH for indicated communities. Bottom: allocentric speed of mouse center in motifs organized by community for female and male *App*^NL-G-F^ mice and WT controls. Relative to sex-matched controls, female, but not male, *App*^NL-G-F^ mice performed ambulatory motifs with excessive allocentric speed. Red indicates disease-affected motifs. (D) Hierarchical organization of motifs and subjects based on motif use relative to sex-matched WT littermates (*n* = 32; 18 *App*^NL-G-F^ and 14 WT mice). *App*^NL-G-F^ and WT mice exhibited different clustering (*p* = 0.0015 by Mann-Whitney rank-sum test). (E) Percentage of time spent in identified communities in 5-min bins during 25 min of exploration in an open arena. Community usages were strongly modulated by time (experience), and *App*^NL-G-F^ mice exhibited impaired time-dependent responses. *p* values were determined by repeated one-way ANOVA. Values are mean ± SEM.

To leverage information about motif organization, we used hierarchical clustering of motif-motif transitions to identify higher-order behaviors. These analyses revealed a distinct hierarchical organization among the 30 motifs, which was used to cluster the motifs into 11 behavioral communities (Figures 4A and S2) according to total usage and the likelihood of sequential use (as opposed to pose similarity). The probability matrix of motif-motif transitions consistently showed that motifs within each community formed distinct clusters (Figure 4B), demonstrating highly structured behavioral dynamics. Notably, motifs of the same community were similarly affected by *App* (Figure 4C, top), validating the concept that communities represent cohesive behaviors. Communities were generically labeled as groom, rear, walk, run, walk-up, fast- and slow-explore, sit-down, and sit-up (see STAR Methods for definition and dendrogram cost function).

Although the VAME training data included only egocentric coordinates (relative to mouse center) without allocentric speed information, communities displayed distinct allocentric speeds consistent with the represented behaviors. Female, but not male, *App*^NL-G-F^ mice moved faster than sex-matched WT controls, particularly in the communities walk, run, and fast and slow-explore (Figure 4C, bottom). Thus, clustering postural motifs into behavioral communities provides an interpretable and ethologically relevant organization of mouse behavior in the context of disease-associated alterations.

To assess the distribution of genotypes and interindividual variability of mice, we also clustered motifs and mice hierarchically based on motif usage. We detected significant differences in clustering between *App*^NL-G-F^ and WT mice (*p* = 0.0015, Mann-Whitney rank-sum test) (Figure 4D), indicating abnormalities in the overall motif use profile. *App*^NL-G-F^ mice also showed a well-defined gradient of behavioral impairment, indicating that spontaneous behavioral changes capture interindividual variability.

### Experience-dependent modulation of behavioral motifs and communities is altered in *App*^NL-G-F^ mice

Next, we investigated whether motif and community use is modulated by time and genotype as mice gain experience during their 25-min exploration of the open arena. We found that motifs and communities were strongly modulated by time and genotype. Interestingly, communities that show habituation (decreased use over time) in WT mice (B-walk, C-run, and E-fast-explore) were underrepresented in *App*^NL-G-F^ mice, whereas communities that showed sensitization (increased use over time) in WT mice (A^IV^-rear, F-slow-explore, and H-sit-up) were overrepresented in *App*^NL-G-F^ mice (Figure 4E). Similar results were found at the motif level (Figure S3B). Thus, *App*^NL-G-F^ mice displayed abnormal habituation and sensitization responses to the environmental context in virtually all identified behaviors. Similar habituation and context-dependent deficits have been observed in APP transgenic and *App* KI mice, as judged by total distance traveled in the OF test.^16,24,27^

### Network analysis of motif transitions reveals increased randomness and altered behavioral sequences in *App*^NL-G-F^ mice

**Figure 5.**
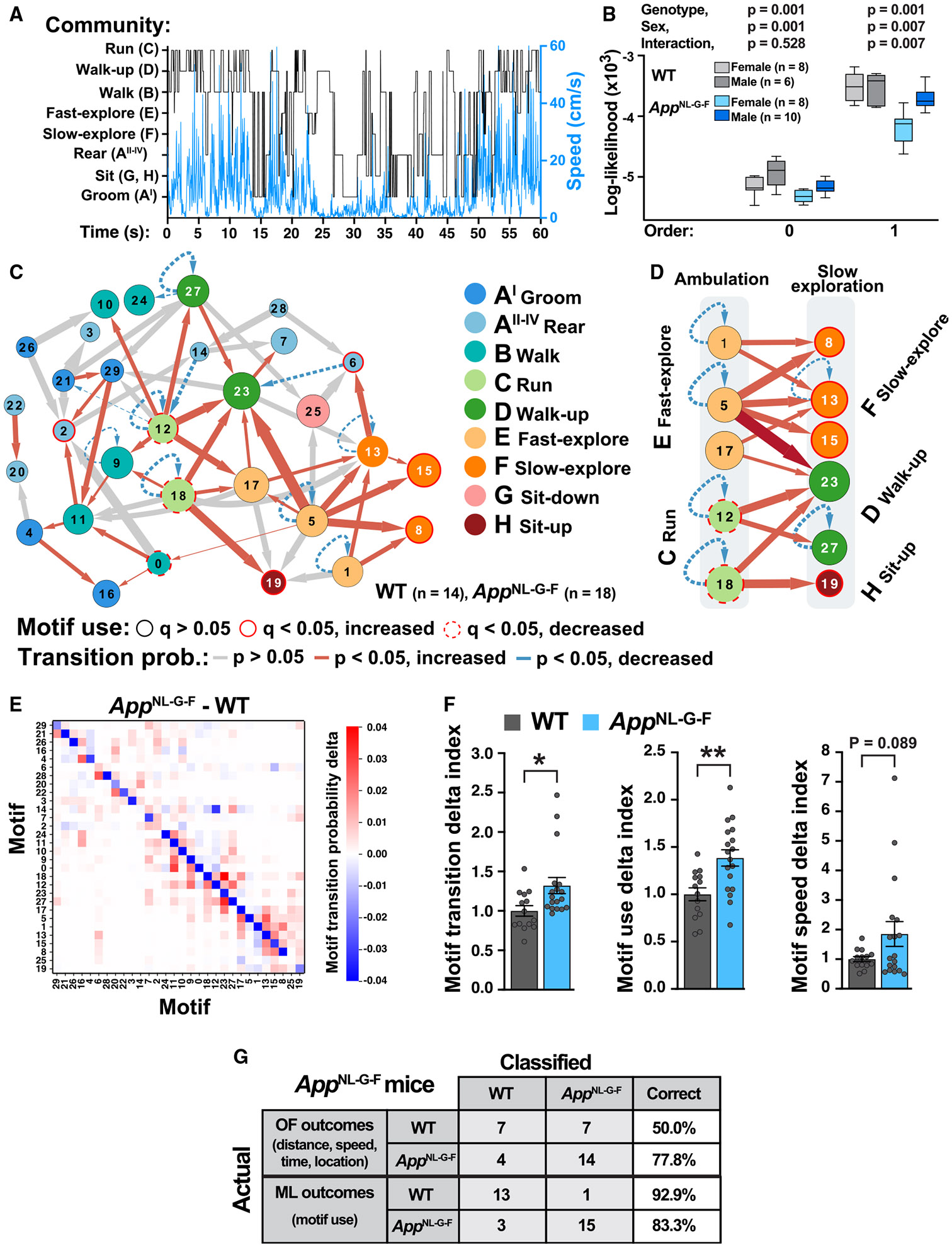
Deconstructing full sequences of spontaneous behavior reveals disorganized behavioral sequences, increased motif transitions, and randomness in *App*^NL-G-F^ mice Full behavioral sequences of motif transitions in 13-month-old *App*^NL-G-F^ mice (*n* = 18; eight females and 10 males) and WT littermate controls (*n* = 14; eight females and six males) were annotated at 25 Hz during 25 min of open-arena exploration (~37,500 potential transitions per mouse) and analyzed with the discrete Markov chain model. (A) Community transition sequences (black) and center speed (blue) of an *App*^NL-G-F^ mouse during 60 s of behavioral exploration. (B) Leave-one-out log likelihood estimates of orders 0 and 1 for the Markov chain model of community transitions. *App*^NL-G-F^ mice, particularly females, had less predictable behavior. *p* values were determined by two-way (genotype and sex) ANOVA. (C and D) Cytoscape network visualization of the top 20% most probable motif transitions depicting topological proximity of associated motifs and communities (color code) and significant alterations of motif use (circular borders) and motif transitions (arrows) in *App*^NL-G-F^ mice. *App*^NL-G-F^ mice had increased transitions (red arrows) and reduced dwell time in motifs (blue dashed arrows) (C). *App*^NL-G-F^ mice systematically favored transitions from fast ambulatory to slow exploratory communities (D). (E) Transition difference matrix depicting the delta probability of transitions between *App*^NL-G-F^ and WT mice. Blue and red indicate reduced or increased probability of transition, respectively, in *App*^NL-G-F^ mice relative to controls. (F) Delta indices (*App*^NL-G-F^ WT mice) of motif transition probability, motif use, and motif speed. **p < 0.01, *p < 0.05 by t test. (G) Classifier analyses (logistic regression) of ML outcomes (motif use) versus conventional open field (OF) outcomes (distance, speed, time, and location) obtained from the same videos in *App*^NL-G-F^ mice. ML outcomes were more sensitive and specific than conventional outcomes. Values are mean ± SEM.

Deconstructing the full sequence of motif-motif transitions may ultimately provide a more sensitive measure of brain function than unitary motif analyses (Figures 2, 3, and 4). To address the overall structure of full behavioral sequences, we used the discrete Markov chain model to assess the probability of motif transitions during 25 min of exploration in the open arena (Figure 5A). This model assesses stochastic infinite sequences of events (e.g., motifs) in which the probability of each event depends on previous events.^29,30^ To assess the predictability of behavior, we fitted Markov chain models at a range of orders (0–3; 640-ms timesteps) (Figure 5B and Table S1).^30^ In *App*^NL-G-F^ mice, particularly females, behavioral sequences were less predictable than in WT mice at all orders, indicating increased entropy or randomness in behavioral organization.

To assess the probability of transitions between motifs, we used Cytoscape^31^ to generate an unbiased network visualization and graphed the top 20% most probable transitions between motifs at the cohort level (*n* = 32 mice). As expected from the transition probability matrix (Figure 4B), we found that motifs were well organized into communities (Figures 5C and 5D). Interestingly, ambulatory behaviors were the hub around which the rest of the behaviors were arranged. *App*^NL-G-F^ mice had substantial increases in the probability of transitions, particularly between motifs of different communities, resulting in decreased dwell time in ambulatory hub motifs (Figure 5C). Notably, *App*^NL-G-F^ mice prematurely transitioned from ambulatory communities (fast exploration or running) to slower exploratory behaviors (walk-up, sit-up, or slow exploration) and were more likely to engage in this behavioral sequence than WT controls (Figure 5D).

Finally, we calculated motif delta indices to score the aggregated motif differences between *App*^NL-G-F^ and WT mice with regard to motif transition probability, motif use, and motif speed. We found that *App*^NL-G-F^ mice had significant abnormalities in delta indices of motif transitions and motif use, and there was a trend toward impaired motif speed delta index (Figures 5E and 5F). Overall, full behavioral sequence analyses revealed significant alterations in the organization of behavioral structure in *App*^NL-G-F^ mice, characterized by increased transitions and randomness, and premature escape of fast ambulatory behaviors to engage in slow and static exploration.

### Spontaneous behavioral alterations in 5xFAD mice are rescued by blocking fibrinogen-microglia interactions

**Figure 6.**
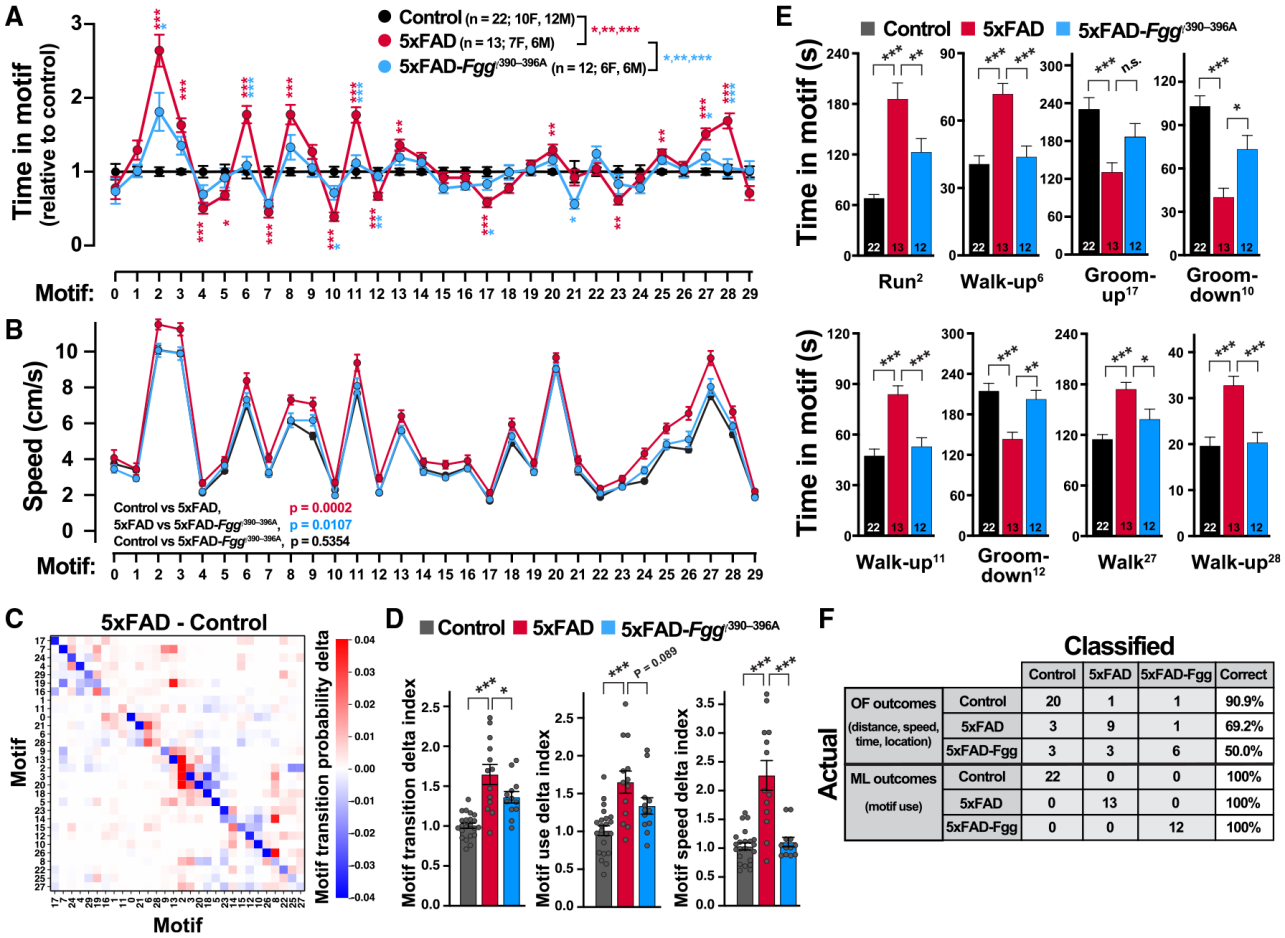
Pronounced 5xFAD-dependent alterations in spontaneous behavior are prevented by blocking fibrinogen-microglia interactions in 5xFAD-*Fgg*^γ390–396A^ mice Spontaneous behavior of 8- to 10-month-old 5xFAD mice (*n* = 13; seven females and six males), 5xFAD-*Fgg*^γ390–396A^ mice (*n* = 12; six females and six males), and controls (*n* = 22; 10 females and 12 males) was recorded for 60 min in a circular open arena. The control group included WT (*n* = 13; six females and seven males) and *Fgg*^γ390–396A^ (*n*=9; four females and five males) mice, as their behavior did not differ (Figure S4A). See Figure S4B for sex effects. (A) Motif use of 30 VAME-identified motifs for all mice. Relative to controls, 5xFAD mice displayed motif use alterations in 17 motifs (red asterisks). Relative to 5xFAD mice, 5xFAD-*Fgg*^γ390–396A^ showed significant improvements in many motifs (blue asterisks). ***q < 0.001, **q < 0.01, *q < 0.05 by FDR-BH. F, female; M, male. (B) 5xFAD mice performed motifs at higher speeds, which was prevented in 5xFAD-*Fgg*^γ390–396A^ mice. Genotype and motif effects were determined by two-way ANOVA (*p* values). (C) Motif transition subtraction matrix depicting the delta probability of transitions between 5xFAD and control mice. Blue and red indicate reduced or increased probability of transition, respectively, in 5xFAD mice relative to controls. (D) Delta indices of motif transition probability, motif use, and motif speed (*n* = 47 mice; 13 5xFAD, 12 5xFAD-*Fgg*^γ390–396A^, and 22 control mice). ***p < 0.001, *p < 0.05 by one-way ANOVA and Bonferroni *post hoc* test for multiple comparisons. (E) Time in motif (seconds) by genotype for the eight 5xFAD-affected motifs restored in 5xFAD-*Fgg*^γ390–396A^ mice (A, *q* < 0.05 by FDR-HB). ***p < 0.001, **p < 0.01, *p < 0.05 by one-way ANOVA and Bonferroni *post hoc* test for multiple comparisons. (F) Classifier analyses (logistic regression) of ML outcomes (motif use) versus conventional OF outcomes (distance, speed, time, and location) obtained from the same videos. ML outcomes were more sensitive and specific than conventional outcomes. Values are mean ± SEM.

The blood coagulation factor fibrinogen is deposited in the brain of patients with AD and is a key mediator of neurotoxic microglia polarization and cognitive impairment in mouse models of AD.^21,22,32,33^ To define spontaneous behavior alterations in a second AD model and determine whether these alterations are sensitive to therapeutic intervention, we studied 8- to 10-month-old 5xFAD mice expressing WT fibrinogen or a mutant fibrinogen (*Fgg*^γ390–396A^) that lacks the inflammatory CD11b receptor-binding site and thus prevents CD11b receptor-mediated interactions with microglia.^22,33^ *Fgg*^γ390–396A^ reduces AD-related pathogenesis, including amyloid burden, dystrophic neurites, synaptic loss, microgliosis, and neuroinflammation in 5xFAD-*Fgg*^γ390–396A^ mice relative to 5xFAD mice.^21,22,34^ We found that 5xFAD mice had prominent ML behavioral alterations in 17 of 30 motifs (Figure 6A). Remarkably, nearly all of these 5xFAD-dependent alterations were ameliorated in 5xFAD-*Fgg*^γ390–396A^ mice, indicating global restoration of spontaneous behavior, including motif use and speeds (Figures 6A and 6B).

We also calculated delta indices for motif transitions, motif use, and motif speed to score the aggregated differences between 5xFAD, 5xFAD-*Fgg*^γ390–396A^, and control mice. We found that 5xFAD-dependent abnormalities in the motif transition and motif speed delta indices were ameliorated in 5xFAD-*Fgg*^γ390–396A^ mice (Figures 6C and 6D). Direct comparisons of the control groups, WT (*Fgg*^+/+^), and *Fgg*^γ390–396A^ mice revealed no significant differences in the usage of any of the motifs (Figure S4A). Thus, *Fgg*^γ390–396A^ does not change spontaneous behavior in a non-AD background. No sex effects were seen in 5xFAD and 5xFAD-*Fgg*^γ390–396A^ mice (Figure S4B). Overall, this global restoration of spontaneous behavior shows that fibrinogen contributes to AD-related behavioral alterations and that ML alterations are susceptible to therapeutic intervention.

**Figure 7.**
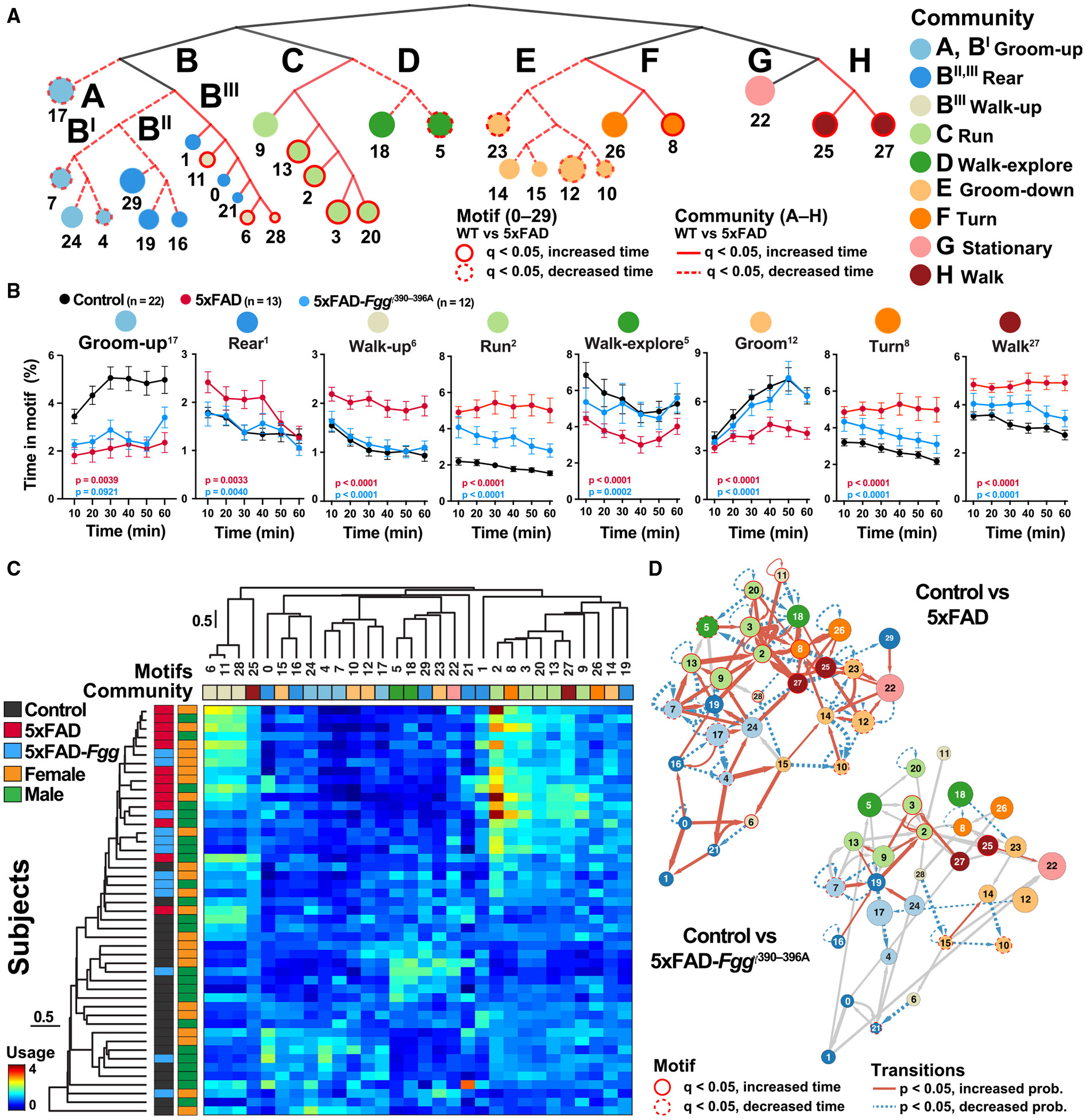
Pronounced 5xFAD-dependent alterations in community usage and motif transition networks are prevented in 5xFAD-*Fgg*^γ390–396A^ mice (A) Hierarchical organization of motifs into communities based on motif transitions and motif use at the cohort level (*n* = 47 mice; 13 5xFAD, 12 5xFAD-*Fgg*^γ390–396A^, and 22 control mice). Node size is proportional to motif use. Significant differences (indicated in red) between 5xFAD and control mice were determined by FDR-BH for 30 motifs and nine communities. (B) Time in motif (%) of a representative motif for each disease-affected community (A) during the 60 min of exploration in an open arena. 5xFAD mice exhibited profound deficits, which were largely prevented in 5xFAD-*Fgg*^γ390–396A^ mice. *p* values were determined by repeated one-way ANOVA with Bonferroni *post hoc* test for multiple comparisons. Values are mean ± SEM. (C) Hierarchical organization of motifs and mice based on motif use relative to sex-matched control littermates. (D) Cytoscape was used to visualize the top 20% most probable motif transitions to depict topological proximity of associated motifs and communities (color code) and significant alterations of motif use (borders) and motif transitions (arrows) for control versus 5xFAD mice (top) and control versus 5xFAD-*Fgg*^γ390–396A^ mice (bottom).

Kinematic and video analyses of the motifs that were significantly improved in 5xFAD-*Fgg*^γ390–396A^ mice were assigned to the following generic behavioral categories: run (motif 2), walkup (motifs 6, 11, and 28), groom-up (motif 17), groom-down (motifs 10 and 12), and walk (motif 27) (Figures 6E and S5). These categories were confirmed by the hierarchical behavioral dendrogram, which showed significant 5xFAD-dependent usage alterations in eight of nine identified behavioral communities (FDR-BH, *q* < 0.05) (Figure 7A, red branches). Furthermore, in a representative selection of 5xFAD-affected motifs (Figure 7B), motif use over time was strongly modulated by genotype, and 5xFAD-*Fgg*^γ390–396A^ mice largely showed full or partial recovery to performances seen in control mice (*p* < 0.05, two-way ANOVA). Hierarchical clustering of motif usage and mice also revealed prominent disease-associated differences (Figure 7C). Together, these results indicate that fibrinogen is a key mediator of behavioral abnormalities in 5xFAD mice. They also suggest that alterations in spontaneous behavior detected by VAME are responsive to AD-related therapeutic interventions and provide unbiased and sensitive outcomes for assessment of treatments.

### Behavioral transition network analysis reveals therapeutic restoration in 5xFAD-*Fgg*^γ390–396A^ mice

To determine whether 5xFAD mice have disease-associated alterations of motif transitions, we generated transition networks at the cohort level for 5xFAD and 5xFAD-*Fgg*^γ390–396A^ mice relative to controls (Figure 7D). As in *App*^NL-G-F^ mice (Figure 5C), motifs were well organized into communities, and ambulatory behaviors (run, walk) formed the hub of the transition network. 5xFAD mice had marked abnormalities in the probability of most transitions, and the transition network was characterized by increased transitions between motifs and reduced probability of dwelling in the same motif (self-transitions). Thus, both *App*^NL-G-F^ and 5xFAD mice exhibit behavioral instability, suggesting that the abnormalities in behavioral structure are an early preclinical feature of AD-related pathogenesis that progresses during clinical stages. To determine whether the motif transition network can be restored by blocking fibrinogen-microglia interactions, we compared 5xFAD-*Fgg*^γ390–396A^ mice and controls. We found that most of the 5xFAD-dependent alterations in transitions were rescued or greatly mitigated in 5xFAD-*Fgg*^γ390–396A^ mice (Figure 7D), indicating restoration of behavioral sequences.

### Classifier analyses reveal VAME outcomes provide better sensitivity and specificity than conventional behavioral outcomes

To directly compare the specificity and sensitivity of VAME to those of conventional behavioral outcomes, we performed classifier analysis (logistic regression) using VAME outcomes (motif usage) or conventional open field (OF) outcomes (distance, speed, time, and location) obtained from the same videos (Figure S6). The genotype classifier using VAME outcomes had a specificity (WT classified as WT) of 92.9% and a sensitivity (*App*^NL-G-F^ classified as *App*^NL-G-F^) of 83.3%, whereas the genotype classifier using conventional OF outcomes had much lower specificity (50.0%) and sensitivity (77.8%) (Figure 5G). Similarly, for all genotypes, VAME outcomes for the 5xFAD cohort also had better sensitivity and specificity than conventional outcomes, categorizing 100% of mice correctly (Figure 6F). Thus, VAME analysis is more sensitive and specific than conventional outcomes for *App*^NL-G-F^ and 5xFAD mice. Notably, only the VAME outcomes differentiated 5xFAD-*Fgg*^γ390–396A^ from control mice, suggesting caution in interpreting therapeutic interventions in mouse models, as conventional approaches may not fully capture remaining disease phenotypes in rescued mice. Finally, to compare our VAME approach with keypoint-MoSeq,^35^ we performed a side-by-side comparison using the same *App*^NL-G-F^ and WT behavioral videos. Although VAME and keypoint-MoSeq used two different algorithms to segment the behavior into motifs or syllables, both approaches identified behavioral alterations in *App*^NL-G-F^ mice (Figure S7).

## DISCUSSION

We studied spontaneous behavior in humanized *App*^NL-G-F^ and 5xFAD mice using the ML-based VAME segmentation platform (https://github.com/EthoML/VAME).^7^ We found that *App*^NL-G-F^ mice exhibit robust age-dependent impairments in spontaneous behavior, including motif utilization, disorganized behavioral sequences, increased transitions, and randomness, indicating that these behavioral alterations reflect disease progression. Spontaneous behavior was altered in both male and female *App*^NL-G-F^ mice, but females were particularly vulnerable to changes in motif usage, motif speed, and randomness of behavioral sequences. 5xFAD mice had more pronounced abnormalities in spontaneous behavior than controls and *App*^NL-G-F^ mice, indicating a gene-dose-dependent effect. Interestingly, both *App*^NL-G-F^ and 5xFAD mice displayed overlapping abnormalities, including increased motif speed, a higher number of transitions, and reduced motif dwell time. These results suggest that these distinct mouse models share common mechanisms underlying AD-related pathogenesis. These results also indicate that AD-related spontaneous behavioral alterations do not require APP overexpression and are likely linked to the pathologically elevated levels of amyloid in *App*^NL-G-F^ mice.^16,19^ We also tested the role of neuroinflammation in AD-related spontaneous behavioral alterations by blocking fibrinogen-microglia interactions in 5xFAD-*Fgg*^γ390–396A^ mice. Notably, the organization of spontaneous behavior was largely restored in 5xFAD-*Fgg*^γ390–396A^ mice, indicating that AD-related spontaneous behavioral changes are sensitive to therapeutic interventions and driven by neuroinflammation.

### Spontaneous behavioral alterations and AD pathogenesis

A major question is whether behavioral alterations in spontaneous behavior are mechanistically related to AD pathogenesis and are therefore driven by the same biological pathways and mechanisms of cognitive decline. Several observations suggest that this is the case. First, *App*^NL-G-F^ and 5xFAD mice shared behavioral alterations (e.g., increased motif speed, elevated number of transitions, and reduced dwell time in motifs), suggesting that these deficits are caused by common mechanisms of disease pathogenesis, such as familial AD mutations or abnormally high amyloid levels. It is worth noting that *App*^NL-G-F^ and 5xFAD mice, in addition to differing in being knockin and transgenic models, have distinct sets of familial AD mutations and patterns of amyloid deposition.^16,36,37^ Second, spontaneous behavioral abnormalities were age dependent and therefore tracked the progression of AD-related pathological and cognitive alterations.^16,19,23^ Third, and more importantly, spontaneous behavioral abnormalities in 5xFAD mice were prevented by an AD-related therapeutic intervention, *Fgg*^γ390–396A^, that prevents CD11b receptor-mediated interactions with microglia^22,33^ and reduces AD-related pathogenesis, including amyloid burden, dystrophic neurites, synaptic loss, microgliosis, and neuroinflammation.^21,22,34^ Thus, our findings suggest that abnormalities in spontaneous behavior are mechanistically related to AD pathogenesis and may offer mechanistically relevant endpoints for assessing AD-related pathways and therapeutic interventions in mouse models.

Although we did not test other neurodegenerative models to assess the disease selectivity of the motif abnormalities, we hypothesize that the structure of spontaneous behavior (e.g., transition networks) will exhibit selective or even pathognomonic elements for different disorders (e.g., AD versus Parkinson’s disease models). Overall, unbiased and high-throughput ML approaches such as the one we used to assess spontaneous behavior address a major unmet need in translational research by identifying translationally relevant, functional outcome measures in AD preclinical models. The current approach does not directly assess cognitive functions, but our findings suggest that these outcomes capture key elements of AD pathogenesis, such as inflammation, that could complement traditional task-oriented behavioral approaches.

Behavioral or neuropsychiatric changes, such as depression, agitation, anxiety, apathy, irritability, delusions, or sleep disruptions, are often apparent years before cognitive decline or dementia. These behavioral changes are associated with increased risk of dementia and AD,^13,14^ suggesting that they contribute to AD pathogenesis.^13,14^ ML approaches have been developed to predict these behavioral abnormalities in AD patients by using data on sleep patterns and physical activity collected by actigraphy from wearable devices.^38^ It is important to emphasize that our results and conclusions apply solely to mouse models of AD and that our ML pipeline currently has no clinical application. However, ML approaches to assess spontaneous behavior in humans, as we have done in mice, can provide early functional outcomes for disease diagnosis and the assessment of treatments and have the potential to reveal and quantify AD-related behavioral manifestations that are otherwise difficult to observe.

### Fibrinogen blockade protects from neurobehavioral abnormalities

Our findings show that VAME is a high-throughput approach for unbiased assessment of therapeutic interventions. In patients and mouse models of AD, fibrinogen extravasates in the brain at sites of impaired blood-brain barrier integrity and cerebrovascular dysfunction, which are early features of AD pathogenesis.^21,39-42^ Fibrinogen is converted to fibrin, which is deposited at sites of microglia activation in the AD brain. Fibrin is present at increased levels in *APOE4* AD patients, correlates with the severity of cerebral amyloid angiopathy, and is detected in amyloid-related imaging abnormalities.^32,43,44^ The binding of Aβ to fibrinogen inhibits fibrin degradation, resulting in chronic fibrin deposition in the AD brain.^32^ Biomarker studies further support the notion that fibrinogen elevation in plasma and cerebrospinal fluid is an early biomarker of dementia risk and is associated with progression of cognitive decline in AD.^39,45-48^ Notably, we found that genetic blockade of fibrinogen-microglia interactions in 5xFAD-*Fgg*^γ390–396A^ mice reduced virtually all the motif usage abnormalities found in 5xFAD mice. These findings, coupled with the restoration of the impaired transition networks in 5xFAD-*Fgg*^γ390–396A^ mice, suggest that fibrinogen is a key driver of the disorganization of behavioral sequences in 5xFAD mice. Our results are consistent with the reduced expression of neuro-degenerative and oxidative stress genes in microglia and protection against cognitive impairment in 5xFAD-*Fgg*^γ390–396A^ mice.^21,22^ Accordingly, a fibrin-targeting antibody that selectively inhibits microglia activation without adverse anticoagulant effects protects from neurodegeneration and suppresses AD-related gene networks in 5xFAD mice.^28,34^ Fibrin-targeting immunotherapy is currently in phase 1 clinical trials.^49^

### ML approaches for behavioral segmentation

ML approaches for behavioral segmentation have the potential to detect subtle or overlooked behavioral abnormalities linked to early brain dysfunction. For example, MoSeq has uncovered previously undetected patterns of spontaneous behavior associated with epileptic activity, providing a behavioral biomarker of brain dysfunction.^9^ MoSeq and VAME are based on unsupervised dimensionality reduction methods, which analyze the implicit structure and patterns of postural motion to identify the most relevant features that capture the spatiotemporal variability of the data to define brief stereotypical postural units. MoSeq^6^ and keypoint-MoSeq^35^ use a statistical framework known as autoregressive hidden Markov model (AR-HMM),^6,50,51^ whereas VAME^1,7^ uses deep recurrent neural networks and variational autoencoders, providing two well-established complementary approaches.^7,52,53^ In a side-by-side comparison of VAME and keypoint-MoSeq, these two approaches for behavioral segmentation differed in motif/syllable distributions but both identified robust behavioral alterations in *App*^NL-G-F^ mice.

VAME works with ventral imaging to assess the movement of seven body parts (four paws, nose, center, and tail base) relative to the mouse center (egocentric coordinates). Although VAME segmentation does not incorporate additional variables such as mouse speed, height, or location, motifs with ambulatory kinematics (e.g., run or fast-explore) exhibited higher allocentric speeds than stationary motifs (e.g., groom or sit). This finding suggests that VAME segmentation and egocentric coordinates reflect allocentric speeds. This property might be important when assessing disease models that have locomotor abnormalities, since adding speed into the neural network may bias motif clustering toward differences in speeds among genotypes and dilute other aspects of behavioral features.

### Communities and transition networks for assessing behavioral organization

Clustering motifs into communities provides a hierarchical organization of mouse behavior that incorporates the probability of motif-motif transitions, thereby grouping motifs according to the likelihood of sequential use rather than pose similarity. The resulting communities represent cohesive postural sequences of ethologically relevant behavior. Indeed, motifs in the same communities responded to *App*^NL-G-F^ and 5xFAD in similar ways in both direction and magnitude, suggesting that communities cluster disease-relevant behaviors. The same communities were also apparent in the unsupervised layout of transition networks, a method that relies on the overall topological connectivity among motifs and is not biased by prior community assignments. By creating and calculating global delta indices (transitions, usage, and speed indices), we found that transitions were particularly sensitive to *App*^NL-G-F^ and 5xFAD, suggesting that the core abnormality in AD models is the disorganization of behavioral sequences rather than the total time spent in each behavior. While sex and genotype interactions are difficult to detect and assess with conventional behavioral tests, VAME captured robust differences in the spontaneous behavior of males and females, making it an ideal platform to assess sex-genotype interactions.

### Scalable and sensitive methods for assessing mouse behavior

Scalable, sensitive, and unbiased methods for behavioral phenotyping are essential for basic and translational neuroscience efforts aimed at phenotyping or assessing therapeutic interventions in mouse models of neurodegenerative disease. The ability to scale ML approaches to large cohorts of mice also facilitates well-powered evaluation of treatment effectiveness across sex and dosages. Moreover, when we assessed the specificity and sensitivity of ML (motif usage) and conventional behavior (distance, speed, and location) outcomes from the same behavioral videos, the ML outcomes classified genotypes and treatments with greater specificity and sensitivity. Remarkably, ML, but not conventional, outcomes distinguished rescued 5xFAD-*Fgg*^γ390–396A^ mice from controls, indicating that conventional approaches lack sufficient sensitivity and specificity to capture remaining disease phenotypes in rescued mice, which may result in an overestimation of the beneficial effects of the intervention.

### Limitations of the study

Our findings suggest that ML approaches to assess spontaneous behavior provide a direct, sensitive, and unbiased measure of brain dysfunction induced by AD-related mechanisms in mouse models. A limitation of our study assessing spontaneous behavior is that specific behavioral domains (e.g., cognitive functions) and functional brain topology were not explicitly assessed. Thus, future studies will be needed to mechanistically determine the contribution of distinct behavioral domains, neural systems, or brain regions to the identified spontaneous behavioral changes. For example, our ML approach may be directly applied to study spontaneous behavior during learning and memory tasks. Another limitation of the study is that it does not resolve whether impairments of spontaneous behavior are driven by biological pathways and mechanisms of cognitive decline. It remains to be determined whether some of these outcomes could represent biomarkers of cognitive dysfunction. Despite these unresolved questions, we speculate that unbiased ML approaches will improve translatability of preclinical testing by providing rigorous quantification of disease-induced behavioral alterations to assess the construct and predictive validity of mouse models of neurodegenerative diseases.

## STAR★METHODS

### EXPERIMENTAL MODEL AND STUDY PARTICIPANT DETAILS

#### Mice

*App*^NL–G-F^ mice were obtained from Drs. Takaomi Saido and Takashi Saito (RIKEN Brain Science Institute, Japan).^15^ We studied male and female homozygous *App*^NL–G-F/NL–G-F^ mice expressing humanized Aβ and the familial Alzheimer’s disease (FAD) Swedish (K670N, M671L), Artic (E693G), and Iberian (717, I) mutations on the C57BL/6J background.^15^ To obtain wildtype (WT) littermate controls, we crossed heterozygous *App*^NL–G-F/+^ mice to produce homozygous *App*^NL–G-F/NL–G-F^ mice and *App*^+/+^ (WT) littermate controls of both sexes. Homozygous *App*^NL–G-F/NL–G-F^ mice are referred to as *App*^NL–G-F^ mice. Heterozygous *App*^NL–G-F/+^ mice were not studied.

5xFAD mice were from The Jackson Laboratory (Jax 0087030).^37^ We studied male and female heterozygous 5xFAD mice overexpressing both human amyloid beta precursor protein (APP) with the Swedish (K670N, M671L), Florida (I716V), and London (V717I) FAD mutations and human presenilin 1 (PS1) with the M146L and L286V FAD mutations on the C57BL/6J background.^37^ *Fgg*^γ390–396A^ mice were from Dr. Jay Degen (University of Cincinnati, OH, USA) and bred in the Akassoglou Lab.^54^ Heterozygous 5xFAD mice were crossed with wildtype (WT) mice to produce heterozygous 5xFAD and WT mice on the C57BL/6J background. Homozygous *Fgg*^γ390–396A/γ390–396A^ mice were crossed with 5xFAD-*Fgg*^γ390–396A/γ390–396A^ mice to produce 5xFAD-*Fgg*^γ390–396A/γ390–396A^ and *Fgg*^γ390–396A/γ390–396A^ mice. Homozygous *Fgg*^γ390–396A/γ390–396A^ mice are referred to as *Fgg*^γ390–396A^ mice. Experimental mice of the same sex were group-housed with access to water and food *ad libitum* in a controlled environment with a 12-h light-dark cycle.

#### Mouse cohorts and experimental groups

Young *App*^NL–G-F^ cohort: 5–6-month-old *App*^NL–G-F^ mice (*n* = 13; 7 females and 6 males; mean age, 6.17 ± 0.62 [SD] months) and WT littermate controls (*n* = 11; 8 females and 3 males; 5.98 ± 0.75 months). Middle-age *App*^NL–G-F^ cohort: 11–15-month-old *App*^NL–G-F^ mice (*n* = 18; 8 females and 10 males; mean age, 13.25 ± 1.59 [SD] months) and WT littermate controls (*n* = 14; 8 females and 6 males; 13.27 ± 1.40 months). Young and middle-age *App*^NL–G-F^ cohorts were behaviorally tested in an open arena to assess spontaneous behavior. To assess spatial learning and memory, advanced-age 19–25-month-old *App*^NL–G-F^ mice (*n* = 17; 9 females and 8 males; mean age 21.88 ± 2.18 months) and WT littermate controls (*n* = 22; 12 females and 10 males; 22.42 ± 1.98 months) were tested in the Morris water maze. These three cohorts are referred to as 6-, 13- and 22-month-old *App*^NL–G-F^ mice, respectively. To assess spontaneous behaviors in the 5xFAD-*Fgg*^γ390–396A^ mice, 8–10-month-old 5xFAD mice (*n* = 13; 7 females and 6 males; mean age, 8.78 ± 0.89 months), 5xFAD-*Fgg*^γ390–396A^ mice (*n* = 12; 6 females and 6 males; 9.06 ± 0.85 months), *Fgg*^γ390–396A^ mice (*n* = 9; 4 females and 5 males; 9.32 ± 0.82 months), and WT mice (*n* = 13; 6 females and 7 males; 9.14 ± 1.01 months) were behaviorally tested in an open arena. Because there were no significant differences in motif usage between *Fgg*^γ390–396A^ and WT mice (Figure S4A), these two groups of mice were combined and treated as control group. This cohort is referred to as the 9-month-old 5xFAD-Fgg^γ390–396A^ mice.

Behavioral experiments were performed in sex-balanced time blocks (morning and afternoon) by investigators who were unaware of the genotype of the mice. Sex effects are reported in the manuscript. All mice were bred and housed at the Gladstone Institutes vivarium, a UCSF AAALAC-approved facility. All mouse experiments were approved by the Institutional Animal Care and Use Committee of the University of California, San Francisco, and were conducted in accordance with the NIH guidelines for the care and use of laboratory animals.

### METHOD DETAILS

#### Behavioral experiments

##### Spontaneous behavior in an open arena

Mice were handled and habituated to the chamber and experimenter before testing. Naturalistic behavior was recorded in an acrylic cylindrical chamber (12 inches in diameter and 14 inches high) for 25 (*App*^NL–G-F^ cohort) or 60 (5xFAD-*Fgg*^γ390–396A^ cohort) minutes during the day, avoiding the first and last 2 h of the lights-on period. Male or female mice were tested in alternating blocks to ensure balanced experimental groups throughout the day. The chambers were cleaned with 70% ethanol between trials.

##### Morris water maze

Twenty-two-month-old *App*^NL–G-F^ mice (*n* = 17; 9 females and 8 males) and WT littermate controls (*n* = 22; 12 females and 10 males) were tested in the Morris water maze. As previously described,^27^ the water maze pool (122 cm diameter) contained opaque white water with a 14-cm^2^ platform submerged 1 cm below the surface. Distinct visual cues were placed on the four walls surrounding the maze. For the hidden platform training, mice were trained to locate the hidden platform over 10 sessions (two sessions per day, 3–4 h apart), each with two trials (60 s each, 15 min apart). The platform location remained constant, and entry points were changed semi-randomly between trials. A 24-hour-probe trial of 60 s (platform removed) was performed after the last day of hidden platform training. For the visible platform test, mice were trained to locate a platform marked with a visual cue (black and green pole; 2 × 15 cm) on top of the platform over 3 sessions (each consisting of two trials, 15 min apart). The visible platform was in the same location used in hidden platform training. Latency, speed, and distance were monitored with an EthoVisionXT video-tracking system (Noldus Information Technology). Three *App*^NL–G-F^ mice were excluded from analysis because they failed to locate the visible platform and had combined latencies and distances for the visible training 3 SD above the mean of WT mice.

#### Videography for machine-learning analysis

Mice exploring an open circular chamber were recorded from below in RBG color (*App*^NL–G-F^ cohort) or monochrome (5xFAD-*Fgg*^γ390–396A^ cohort) video at 25 frames per second with a 1.3 MP GigE camera (acA1300-60gc; Basler) and a varifocal lens (4.5–12.5 mm; Computar) using EthoVisionXT (Noldus Information Technology). Four adjustable light bricks (Panel Go; Lume Cube) lit the chambers from below for homogeneous illumination of the paws and other body features. Videos were converted from mpg to mp4 format before machine-learning analysis.

#### Pose estimation

To capture mouse postural movement, we used DeepLabCut (DLC, version 2.1.8.2),^2^ a supervised deep-learning tool which tracks animal posture by using deep convolutional neural networks pretrained on ImageNet.^55^ To create supervised annotations for the *App*^NL–G-F^ cohort, 10 frames were extracted from each of the 32 videos, and 9 body parts (nose, paws, center, tail base, mid-tail, tail tip) were manually labeled in each extracted frame. The network (ResNet-50) was trained up to 10^6^ iterations until the error converged (train error: 1.01 pixels, 0.75 mm; test error: 5.02 pixels, 3.73 mm) and precise virtual skeletons were estimated for all mice. Supervised annotations for the 47 videos of the 5xFAD-*Fgg*^γ390–396A^ cohort were created by using the same approach, with 20 frames from 13 representative videos labeled manually (10^6^ iterations; train error: 1.44 pixels, 1.06 mm; test error: 5.30 pixels, 3.94 mm). Google Colaboratory (Colab Pro GPU) was used for DLC network training, video analysis, and labeled video creation. DLC pose-tracking results were exported to CSV files for further analysis.

#### Behavioral segmentation and sequencing

To study behavioral manifestations in AD mouse models, we further developed our unsupervised behavioral segmentation and sequencing tool, Variational Animal Motion Embedding (VAME).^7^ We used this tool to identify multidimensional clusters of postural motions (motifs) from egocentric spatiotemporal coordinates derived from DeepLabCut pose estimation, thereby permitting calculation of kinematic variables to precisely identify cohesive ethologically-relevant behaviors. For the *App*^NL–G-F^ cohort, custom MATLAB code (version 2021b; MathWorks) was used to align mouse coordinates along the midline for every frame. The midline was defined as a vector from the mouse center to the tail-base and the center of the mouse was defined as the origin (0,0). For the 5xFAD-*Fgg*^γ390–396A^ cohort, pose data was egocentrically aligned using the VAME alignment module using the nose-tailbase axis.^7^ In both cohorts, mid-tail and tail tip coordinates were not included in the VAME training data, as these body parts reflect a time-lagged version of mouse motion. The remaining seven egocentrically-aligned coordinates and associated DLC confidence values for each cohort were used to train separate VAME networks until the test-training loss had converged. We used the standard RNN network as defined as default configuration in the original publication^7^ with the default number of latent features (zdims in the config.yaml = 30), time window size in seconds (time_window in config.yaml = 16 frames or 0.64 s), and number of motif clusters (n_clusters in the config.yaml = 30). Latent information (30 z-dimensions) was used to identify 30 *k*-means- or HMM-clustered motifs in the training data, and each frame was assigned a motif within a sliding time window of 16 frames (±320 ms). Because *k*-means demonstrated higher probability of correct genotype classification using logistic regression compared to HMM (93.8% vs. 90.6%) (Figure S1A), *k*-means was chosen to identify motifs. DeepLabCut-labeled motif videos and community videos were generated for visual inspection of VAME-identified postural motions. VAME outputs associated with *App*^NL–G-F^ mice were exported to MATLAB and a Jupyter notebook for post-processing. VAME outputs associated with 5xFAD-*Fgg*^γ390–396A^ mice were exported to a separate Google Colab notebook for post-processing (see Code Availability for download link).

An independent VAME model was created for each of the two cohorts, to maximize detection of disease-associated alterations observed within these distinct AD mouse models. Therefore, motifs and communities expressed by the respective control/WT animals differ across the two VAME models.

#### Uniform manifold approximation (UMAP)

We used the UMAP dimensionality reduction approach (*umap-learn* Python package) to visualize the topology of latent vectors produced by ethoML for *App*^NL–G-F^ mice. A random sample of 10,000 frames was selected from the full cohort and segmented into corresponding genotype and sex groups to generate comparison manifolds (male vs. female and *App*^NL–G-F^ vs. WT), as well as a UMAP manifold of the respective 30 motifs identified by ethoML for each cohort.

#### Motif and community usage

Motif and community use were defined as the total number of frames or seconds a mouse performed a given motif or community during the naturalistic behavior assay. For *App*^NL–G-F^ mice, differences in motif use between genotypes relative to WT were assessed in sex-matched normalized data by false discovery rate with Benjamini-Hochberg test for multiple comparisons as described.^56^ Significant *App*-affected motifs (*q* < 0.05) were analyzed by two-way ANOVA to assess genotype and sex interactions. Changes in motif and community use over time were assessed by repeated measures one-way ANOVA in 5-min bins. The 5xFAD-*Fgg*^γ390–396A^ cohort was analyzed in the same manner. 5xFAD-affected motifs restored in 5xFAD-*Fgg*^γ390–396A^ mice (*q* < 0.05) were analyzed by one-way ANOVA and Bonferroni post hoc test for multiple comparisons.

#### Motif usage correlation cluster analyses

Correlation cluster analyses were performed on a matrix of motif usages for all mice in a given cohort in order to group motifs that were used to a similar extent, and to rank mice according to their motif usage patterns. We first calculated the Pearson’s correlation between pairs of columns containing each subject’s motif usage relative to the average total usage of that motif by sex-matched control mice, then subtracted each element of the resultant correlation matrix from 1 to obtain the dissimilarity matrix. The *linkage()* and *dendrogram()* MATLAB functions were used to obtain and visualize a hierarchical cluster tree that grouped motifs with similar usage patterns across mice. Correlations between mice with similar motif usage patterns were found according to the same method. By applying this clustering approach to both dimensions of the motif usage matrix, both motifs and mice with similar motif usage patterns were grouped and ordered according to the out permutation order of the *dendrogram()* function.

#### Motif transition matrix

To create the motif transition matrix at the cohort level, we created a 2D histogram of motif-motif transitions observed over all trials. To focus on transitions from one motif to another, the diagonal was set to zero, and each row was normalized to the total number of times a mouse transitioned out of that motif. The resulting asymmetric transition matrix, *T*, was used to determine the motif transition alteration index (TAI) and was used as an input to the hierarchical community dendrogram function.

#### Hierarchical community dendrogram

A hierarchical motif-community dendrogram at the cohort level was generated in a Jupyter notebook with the NetworkX Python library. Using the transition probability matrix and motif usage for the full cohort, we implemented a cost function (Equation 1) to iteratively merge clustered nodes with higher transition probabilities relative to each other.


(Equation 1) $$cost=\text{min}\left(usage_{i}+\frac{usage_{j}}{prob_{i,j}+prob_{j,i}}\right).$$

To identify behavioral communities of associated motifs, we inspected kinematic and speed trends for each motif according to dendrogram-order. For the *App*^NL–G-F^ cohort, communities (A–H) were identified by segmenting the dendrogram at height = 3. Community A had distinct kinematics and genotype effects within the branched clusters and was therefore further segmented into 4 communities (Figures 4A, 4C and S2). For the 5xFAD-*Fgg*^γ390–396A^ cohort, communities (A–H) were similarly identified by segmenting at height = 3. Community B had distinct kinematics and genotype effects within the branched clusters and was therefore further segmented into three communities. Communities A and B^I^ had similar kinematics and together constituted the Groom-up community. Within community B^III^, motifs 1, 0, and 21 were associated with rearing, whereas motifs 11, 6, and 28 were associated with rapid ambulatory wall approach and rear initiation (Figure S5). These two sets of motifs were differentially color-coded and respectively grouped into the B^II^/B^III^ Rear community and the B^III^ Walk-up community.

#### Transition network analyses and visualization

Transition probabilities between motifs revealed important information about the organization of behavior in healthy and diseasemodel mice. We used Cytoscape for network visualization of motif transition trends for *App*^NL–G-F^ mice. Node size represents the non-normalized time in a motif across all mice. Directional edges show the highest 20% of transitions; edge width is proportional to transition probability. Significant differences between genotypes (*App*^NL–G-F^ vs. WT) are indicated at the node level with red borders (solid, overexpressed; dashed, underexpressed) and at the transition level with edge color (solid red, overexpressed; dashed blue, underexpressed). To identify disease-associated alterations in sequential motif transitions, we used a discreet Markov Chain model to identify altered “self-edges” (i.e., probability of staying in a given state).


$$edge\;weight=\frac{transitions_{i,j}}{usage_{i}}$$

This definition satisfies a property of the transition probability matrix such that the sum of transition probabilities out of motif i is equal to 1. It also allows for estimates of the probability that the mouse will return to the same motif. For example, once the mouse starts exhibiting a motif that could be called an “absorbing” state, it would continue to do so for the rest of the observation period; in this case, the transition probability of returning to this motif would be exactly 1.

5xFAD and 5xFAD-*Fgg*^γ390–396A^ mice were analyzed in a similar manner. We found the union of the set of transitions was significantly affected by 5xFAD-affected motifs and significantly restored in 5xFAD-*Fgg*^γ390–396A^ mice. We then used Cytoscape to visualize the highest 20% of transitions in the case of control mice vs. 5xFAD mice (Figure 7D, top), and control mice vs. 5xFAD-*Fgg*^γ390–396A^ mice (Figure 7D, bottom).

For the transition network statistics, we used the false discovery rate with Benjamin-Hochberg multiple testing adjustment. To estimate the change in the log odds (or the log odds ratio) between the two genotypes of transitioning from motif i to motif j, we used the generalized linear mixed effects model (implemented in the *glmer* function, with family argument set to binomial, in the lme4 package in R) with a random effect for each mouse.

#### Evaluation of order of Markov chain models and pseudo Bayes factors

##### Order of Markov chain models

For *App*^NL–G-F^ mice, we defined 11 communities (A^I^, A^II^, A^III^, A^IV^, B, C, D, E, F, G, and H) for each frame over the 25-min period of open arena exploration (see community glossary below) and fit Markov chain models of different orders (0, 1, 2, and 3)^30^ to determine whether behavior exhibited in the recent past could predict future behavior. We evaluated these models in the context where time was discretized in consecutive 640 ms intervals.

The models were evaluated using a Leave-One-Out Cross Validation (LOOCV) framework to prevent overfitting. The LOOCV framework was implemented separately for WT and *App*^NL–G-F^ mice. The parameters (probability or transition probabilities) of the different models were estimated using the observations from all but one mouse and the log likelihood of the sequence of communities exhibited by the held-out mouse was evaluated by using the fitted parameters.

For the order 0 model, the probability of the ith mouse exhibiting community c at any observed 640-ms interval is estimated as

$$p_{c}^{i}=\frac{N_{c}^{i}}{N}$$

where N denotes the total number of 640 ms intervals over the period of observation and $N_{c}^{i}$ denotes the number of intervals where the ith mouse displayed community c. This estimate for all mice but the ith mouse, $p_{c}^{i^{\prime }}$, is given by the sample mean of the probability estimates in these mice:

$$p_{c}^{i^{\prime }}=\frac{\sum_{j=1;j\neq i}^{M}p_{c}^{j}}{M-1}$$

where M is the number of mice in the group. The leave-one-out log likelihood estimate of the order 0 model for the observed sequence of communities in the ith mouse is then given by

$${ℒ}_{0}^{i}=\sum_{c=1}^{11}N_{c}^{i}.\log\;(p_{c}^{i^{\prime }})$$

Transition probabilities underlie the models of order greater than 0. Let s denote a state in these models. The communities themselves would be the states for the order 1 model, ordered pairs of communities would encode states for the order 2 model, and ordered triplets of communities would encode states for the order 3 model. The estimate of the transition probability from state s to community c for the ith mouse, $q_{sc}^{i}$ is given by

$$q_{sc}^{i}=\frac{N_{sc}^{i}}{N_{s}^{i}}$$

where $N_{s}^{i}$ denotes the number of 640 ms intervals mouse i spent in state s. $N_{sc}^{i}$ denotes the number of times mouse i transitions to community c from state s. This estimate for all but the ith mouse, $q_{sc}^{i^{\prime }}$, is similarly estimated by the sample mean. The leave-one-out log likelihood estimate of the order k(k>0) model for the observed sequence of communities in the ith mouse is then given by

$${ℒ}_{k}^{i}=\sum_{s=1}^{S_{k}}\sum_{c=1}^{11}N_{sc}^{i}.\log\;(q_{sc}^{i^{\prime }})$$

###### Pseudo Bayes factors.

The estimate of the log of pseudo Bayes factors^57^ for the Markov chain model comparison between order k and order l is

$$logPBF_{kl}=\sum_{i=1}^{M}({ℒ}_{k}^{i}-{ℒ}_{l}^{i})$$

#### Delta indices for motif transitions, motif use, and motif speed

To compare the impact of disease and therapeutic interventions on ML behavioral measures at the individual and group levels, we created a set of three summary measures (delta indices) that quantify the magnitude of behavioral changes. For visual comparison across the three delta index types in Figures 5F and 6D, individual subject index scores were normalized to the control group’s mean index value for that index type.

The Transition Delta Index (TDI) is a summary measure that quantifies how the motif transition probability matrix differs as a function of genotype group membership. Mathematically, the TDI is the grand sum of the matrix depicting the absolute difference between the transition probability matrix of the ith subject of a particular group and the transition probability matrix of the baseline control group average $\bar{C}$ (Figures 5E and 6C). The TDI is normalized by the inverse of number of possible transitions between m motifs $\left(m^{2}\right)$ to ensure invariance to the number of motifs identified in a given experiment.

The TDI can be formulated by the given expression below,

$$TDI=\sum_{m}\frac{|T_{i}-T_{\bar{C}}|}{m^{2}}$$

where,

m is the number of motifs,

$T_{i}$ denotes the transition probability matrix of the ith subject of genotype group, and

$T_{\bar{C}}$ denotes the group-average transition probability matrix of the appropriate baseline control group.

The Use Delta Index (UDI) is a summary measure that quantifies how the motif usages are altered across genotype. Mathematically, it is the grand sum of the matrix depicting absolute difference between an array consisting of the motif usages (in percent) of the $i^{\mathrm{th}}$ subject of a particular genotype and the mean motif usage (in percent) across all motifs for all subjects in the Control group C. This grand sum is further normalized with respect to the total number of motifs identified in a given experiment.

The UDI can be formulated by the given expression below,

$$UDI=\sum_{m}\frac{|U_{i}-U_{\bar{C}}|}{m}$$

where, m is the number of motifs

$U_{i}$ denotes the of the $i^{\mathrm{th}}$ subject of the genotype group

$U_{\bar{C}}$ denotes the mean motif usage across all motifs for all subjects in the control group.

The Speed Delta Index (SDI) is a summary measure that quantifies how the motif speeds are altered across genotype. Mathematically, it is the grand sum of arrays depicting the absolute difference between an array consisting of the motif speeds of the $i^{\mathrm{th}}$ subject of a particular genotype and the mean motif speed across all motifs for all subjects in the control group. This grand sum is further normalized with respect to the total number of motifs identified in the given experiment.

The SDI can be formulated by the given expression below,

$$SDI=\sum_{m}\frac{|S_{i}-S_{\bar{C}}|}{m}$$

where

m is the number of motifs

$S_{i}$ denotes the motif speed array of the $i^{\mathrm{th}}$ subject of the genotype group, and

$S_{\bar{C}}$ denotes the mean motif speed across all motifs for all subjects in the Control group.

#### Keypoint-MoSeq

To compare VAME with alternative behavioral sequencing platforms, a separate analysis for the middle-age 13-month-old *App*^NL–G-F^ mice was conducted on the same videos at the University of Alabama at Birmingham in the laboratory of Dr. Erik Robeson, employing keypoint-MoSeq for the independent identification of pose trajectories (i.e., syllables). The pose trajectories were derived from egocentric coordinates obtained through the DeepLabCut pose estimation, which was previously examined using VAME, as detailed earlier. Adhering to the established procedures outlined in the keypoint-MoSeq repository (https://github.com/dattalab/keypoint-moseq/), we executed the standard workflow to predict syllables for each video frame. The supercomputing cluster at the University of Alabama at Birmingham (A100 GPUs) was used for keypoint-MoSeq training and analysis.

For egocentric alignment, the anterior reference point was determined using the nose, while the posterior reference point was identified as the base of the tail. A hyperparameter, kappa, was configured at a value of 10000 to ensure a comparable syllable length to the output generated by VAME. Notably, during the analysis, the middle of the tail and the tail tip were excluded from both training and subsequent analysis.

To create a model in keypoint-MoSeq, pose trajectories were derived from a Principal Component Analysis (PCA) of the egocentrically-aligned key point time series extracted from DeepLabCut. Subsequently, an autoregressive hidden Markov model (AR-HMM) was fitted to the pose trajectory data over 50 iterations. Finally, a complete keypoint-MoSeq model was created using the provided documentation and trained for 500 iterations. Keypoint-MoSeq outputs including individual trial data, cumulative data frames, and statistical summaries were sent to Gladstone for statistical analysis.

For the side-by-side comparison between VAME and keypoint-MoSeq, we created independent models. Thus, the motifs in Figure S7 are different from those in the rest of the manuscript.

#### Community glossary

The VAME probabilistic algorithm assigned a motif to each frame by determining the motif with the dominant likelihood given the eight previous and following frames. We therefore note that VAME community videos contain some flexibility in behavioral expression. Glossary definitions represent the most dominant behavior and were determined by observing community videos and kinematics (Figure S2), which reveal ethologically-relevant behaviors not obvious to human observers. For *App*^NL–G-F^ mice, we defined the 11 identified communities as described below:

A^I^, Groom. Grooming behavior. Mouse is stationary, sometimes sitting back on its hind legs, and bends neck down then rapidly moves paws over ears and nose with head movement toward the mouse’s right (Video S1).

A^II^, Rear: Rearing behavior. Fully extended rearing posture, supported against the chamber wall with head movement to the mouse’s left (Video S2).

A^III^, Rear: Rearing behavior. Supported rearing against the chamber wall with body curvature to the mouse’s right (Video S2).

A^IV^, Rear: Rearing behavior. Partially to fully extended rearing posture, not supported by the chamber wall. Motifs observed show the initiation of an unsupported rear and subsequent extension to a full rear (Video S2).

B, Walk: Slow locomotor ambulation behavior. Walks across the center of the chamber or circles the chamber perimeter. Motifs observed demonstrate the initiation of slow movement with the head extending outward in no particularly consistent direction (Video S3).

C, Run: Fast locomotor ambulation behavior. Motifs observed show rapid movement along the arena perimeter (Video S4).

D, Walk-up: Exploratory behavior. Slowly taking a few steps forward and sniffing, often with one or two lifted front paws and a slight raise of the torso and head (Video S5).

E, Fast-explore: Exploratory behavior. Moving forward with side-to-side sweeping of the head, neck angled up. Motifs observed demonstrate return from a raised posture and rapid forward ambulation (Video S6).

F, Slow-explore: Exploratory behavior. Slow forward movement interrupted by pauses, sniffing upwards, and neck extensions (Video S7).

G, Sit-down: Sitting on rump with rounded back, with side-to-side nose movement and forward head extension (Video S8).

H, Sit-up: Sitting back on hindlimbs (hindlimbs vertically aligned with center) and resting without full extension into an unsupported rear (Video S9).

#### Histology and immunohistochemistry

*App*^NL–G-F^ mice were anesthetized and transcardially flush-perfused with 0.1 M phosphate buffer (PB), and brains were extracted and drop-fixed in 4% phosphate-buffered paraformaldehyde at 4°C for 48 h. After rinsing with PB saline (PBS), brains were transferred to 30% sucrose in PBS at 4°C for 24 h and coronally sectioned with a sliding microtome. Ten subseries of floating sections (30 mm) were collected per mouse and kept at −20°C in cryoprotectant medium until use. Each subseries contained sections throughout the rostrocaudal extent of the forebrain. Brain sections were washed 3 times for 10 min with PBS to remove cryoprotectant medium and once with 0.5% Triton X-100 (PBTx) to permeabilize the tissue. Endogenous peroxidases were blocked with a 15-min incubation with 3% hydrogen peroxide and 10% methanol in PBS. Sections were subsequently washed three times for 10 min each in PBSTx. Nonspecific binding was blocked with a blocking solution containing 10% normal donkey serum (Jackson ImmunoResearch, 017-000-121) and 0.2% gelatin (Sigma-Aldrich, G2500) in PBSTx for 1 h. Brain sections were incubated overnight with biotinylated 82E1 mouse anti-Aβ antibody (IBL, 10326) at 1:250 dilution for staining or rabbit anti-Iba1 antibody (Wako, 019–19741) in 3% normal donkey serum 0.2% gelatin and PBSTx at 4°C. For anti-Iba1 staining, after washes, brain sections were incubated with biotinylated donkey anti-mouse antibody (Jackson Labs) at 1:500 dilution for 2.5 h at room temperature. Unbound detection antibodies were removed with three washes of 10 min with 0.5% PBSTx and one wash with PBS for 10 min. Iba1 and 82E1 stainings were developed with an avidin-biotin complex (ABC) kit (Vector Laboratories, PK-6100) according to manufacture protocol and sections were incubated for 1 h at room temperature. Brain sections were subsequently washed three times for 10 min each with PBS at room temperature and incubated with diaminobenzidine (DAB; Vector Labs) for colorimetric development. Sections were then washed three times for 10 min each with PBS and mounted in 1.2% gelatin in H_2_O and allowed to dry. After two 5-min washes in xylene, brain sections were permanently mounted on slides and coverslipped for analysis.

### QUANTIFICATION AND STATISTICAL ANALYSIS

#### Statistical analysis

Statistical analyses and graphs were done with SPSS (28.0.1.1), MATLAB (2018b), or Prism 10. Statistical tests and p or q values are indicated in the figure or figure legends. Briefly, false discovery rate (FDR) with Benjamin-Hochberg correction for multiple comparisons (q values) was used to assess genotype effect on motif or community usage or speeds among all motifs and communities. two-way ANOVA was used to assess sex, genotype, and sex–genotype interaction effects on motif use and log likelihood estimates. Repeated one-way ANOVA was used to assess genotype effects on motif use changes over time. Pseudo Bayes factors was used to assess Markov chain model comparisons of orders and experimental groups. N represents number of mice or frames and is indicated in the figure or figure legends. Values are mean ± SEM. Numbers of males and females per experimental group are indicated in figure legends and text. Null hypotheses were rejected by double-tailed tests with an alpha value of 0.05.

Probability of transitions between motifs for the motif transition networks were calculated by a generalized linear mixed-effects model (GLMM) to model the disease-associated differences in the rate of transitions between every pair of identified motifs in the *App*^NL–G-F^ and 5xFAD cohorts. Specifically, two numbers representing the number of frames showing a transition from the first motif to the second motif in the evaluated pair of motifs and the total number of frames spent in the first motif (in the pair) were recorded for each mouse in the two cohorts. For the *App*^NL–G-F^ cohort, this pair of numbers was modeled by using a GLMM assuming a binomial probability error distribution with a random effect for each mouse and a fixed effect capturing the log odds ratio representing the differences in log odds of transitioning between the first and second motifs for the *App*^NL–G-F^ mice versus the WT mice. The control mice in the 5xFAD cohort included both WT and *Fgg*^γ390–396A^ mice. Since their motif usage did not differ (Figure S4A), these two groups were combined into a single control group. The random effects for the GLMM included an animal model (control, *Fgg*^γ390–396A^, and 5xFAD-*Fgg*^γ390–396A^) effect, a separate mouse-specific effect sampled hierarchically within the animal model, and a fixed effect capturing the disease-specific log odds ratio. The GLMM was implemented by using the *glmer* function in the lme4^58^ package in R.^59^

We performed a binomial (WT vs. *App*^NL–G-F^) or multinomial (control, *Fgg*^γ390–396A^, and 5xFAD-*Fgg*^γ390–396A^) logistic regression classifier analyses to assess the sensitivity and specificity of VAME outcomes (motif use of the 30 motifs; Figures S6G and S6M) and conventional behavioral outcomes in the open field (23 variables including speed, distance, duration in the different arenas; Figures S6A–S6E and S6H–S6L). We used forward stepwise conditional method for variable entry and removal (<0.05) for all mice (no held out group was tested). For WT vs. *App*^NL–G-F^: ML outcomes the model included constant and motifs 6 and 28 (entry order) (correct classification: WT = 92.9% and *App*^NL–G-F^ = 83.3%; AIC = 24.652); conventional behavior outcomes model included middle inactive (correct classification: WT = 50.0% and *App*^NL–G-F^ = 77.8%; AIC = 37.052). For control, *Fgg*^γ390–396A^, and 5xFAD-*Fgg*^γ390–396A^: ML outcomes the model included intercept and motifs 2, 28, 22, 4, and 11 (entry order) (correct classification: control = 100%, *Fgg*^γ390–396A^ = 100%, and 5xFAD-*Fgg*^γ390–396A^ = 100%; AIC = 24.002); conventional behavior outcomes model included intercept and middle distance, wall speed, and center inactive (entry order) (correct classification: control = 90.9%, *Fgg*^γ390–396A^ = 69.2%, and 5xFAD-*Fgg*^γ390–396A^ = 50.0%; AIC = 70.857). For HMM and *k*-means, HMM model included constant and motifs 9, 25, and 10 (entry order) (correct classification: WT = 92.9% and *App*^NL–G-F^ = 88.9%; overall, 90.6%; AIC = 20.029); *k*-means model included constant and motifs 17 and 7 (entry order) (correct classification: WT = 94.4% and *App*^NL–G-F^ = 92.8%; overall, 93.8%; AIC = 34.749).

## Supplementary Material

1

2

4

5

6

7

8

9

10

11

## Figures and Tables

**Table:** KEY RESOURCES TABLE

REAGENT or RESOURCE	SOURCE	IDENTIFIER
Antibodies		
Amyloid β (N) (82E1) Anti-Human Biotin	IBL	RRID: AB_10705565
Iba1	Wako	RRID: AB_839504
Experimental models: Organisms/strains		
*App*^NL–G-F^: C57BL/6-App<tm3(NL-G-F)Tcs>	RIKEN; Dr. Saido	RRID:IMSR_RBRC06344^15^
5XFAD: B6.Cg-Tg (APPSwFlLon, PSEN1*M146L*L286V)6799Vas/Mmjax	Jackson Laboratory;	RRID:MMRRC_034848-JAX^37^
*B6.Fgg* ^γ390–396A^	Dr. Jay Degen (University Of Cincinnati, Cincinnati, OH, USA)	Flick et al.^54^

## Highlights

- VAME reveals robust alterations in spontaneous behavior in *App*^NL-G-F^ and 5xFAD mice
- VAME has higher specificity and sensitivity than conventional behavioral approaches
- Fibrinogen-induced neuroinflammation drives behavioral changes in 5xFAD mice
- VAME outcomes are sensitive to AD-related therapeutic intervention
