# Supplementary material for: Machine learning reveals prominent spontaneous behavioral changes and treatment efficacy in humanized and transgenic Alzheimer’s disease models
Source: Cell Rep. Author manuscript; Available in PMC 2025 Apr 21. (PMC12010505; doi:10.1016/j.celrep.2024.114870)
Supplement: 1 [file NIHMS2067914-supplement-1.pdf]

Cell Reports, Volume *43*

Supplemental information

Machine learning reveals prominent spontaneous behavioral changes and treatment efficacy in humanized and transgenic Alzheimer's disease models

Stephanie R. Miller, Kevin Luxem, Kelli Lauderdale, Pranav Nambiar, Patrick S. Honma, Katie K. Ly, Shreya Bangera, Mary Bullock, Jia Shin, Nick Kaliss, Yuechen Qiu, Catherine Cai, Kevin Shen, K. Dakota Mallen, Zhaoqi Yan, Andrew S. Mendiola, Takashi Saito, Takaomi C. Saido, Alexander R. Pico, Reuben Thomas, Erik D. Roberson, Katerina Akassoglou, Pavol Bauer, Stefan Remy, and Jorge J. Palop

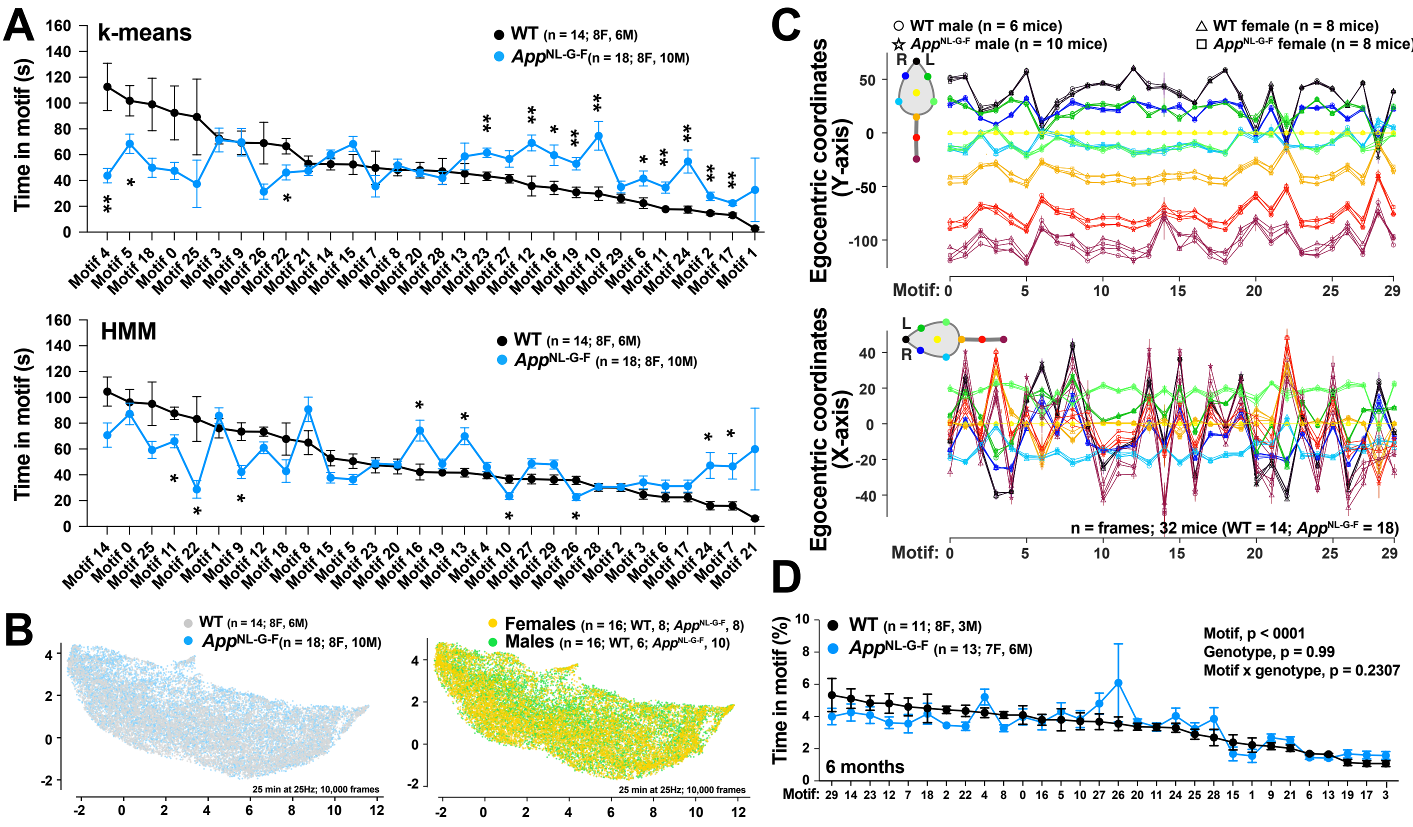

Figure S1, related to Figures 2 and 3. Validations, including comparison of k-means vs HMM segmentation and evidence that VAME postural motifs are consistent across sex and genotypes as shown by UMAP and kinematic validation in *App*$^{\text{NL-G-F}}$ mice.

(A) Side-by-side comparison of k-means (top) and HMM (bottom) algorithms for segmentation of the VAME model embeddings into 30 motifs. k-means demonstrated higher probability of correct genotype classification compared to HMM by logistic regression analyses (93.8% vs. 90.6%) and was chosen to cluster motifs. *q < 0.05 and **q < 0.01 relative to WT with FDR Benjamini-Hochberg correction for multiple comparisons.

(B) UMAP representation of the 30 identified motifs (not color coded) by genotype (left) and sex (right) in 13-month-old *App*$^{\text{NL-G-F}}$ mice (n = 18; 8 females and 10 males) and WT littermate controls (n = 14; 8 females and 6 males). Points in each graph represent 10K frames (5K frames per genotype or sex) randomly selected from the entire cohort and duration of the behavioral session. Motifs were distributed across the UMAP dimensions with no appreciable differences by sex or genotype.

(C). Egocentric postural coordinates in the rostral–caudal (y-axis, top) and left–right (x-axis, bottom) axes of the nine body parts for the 30 identified motifs by genotype and sex in 13-month-old *App*$^{\text{NL-G-F}}$ mice (n = 18; 8 females and 10 males) and WT littermate controls (n = 14; 8 females and 6 males). Points for each body part represent the average location over the first 20 consecutive frames (or 800 ms) for each observation of a motif. Motifs had specific and distinct coordinates regardless of sex and genotype. Values are mean ± SEM.

(D) 6-month-old *App*$^{\text{NL-G-F}}$ mice (n = 13; 7 females and 6 males) and WT littermate controls (n = 11; 8 females and 3 males) did not exhibit ML alterations in spontaneous behavior by false-discovery rate with FDR-BH for multiple comparisons.

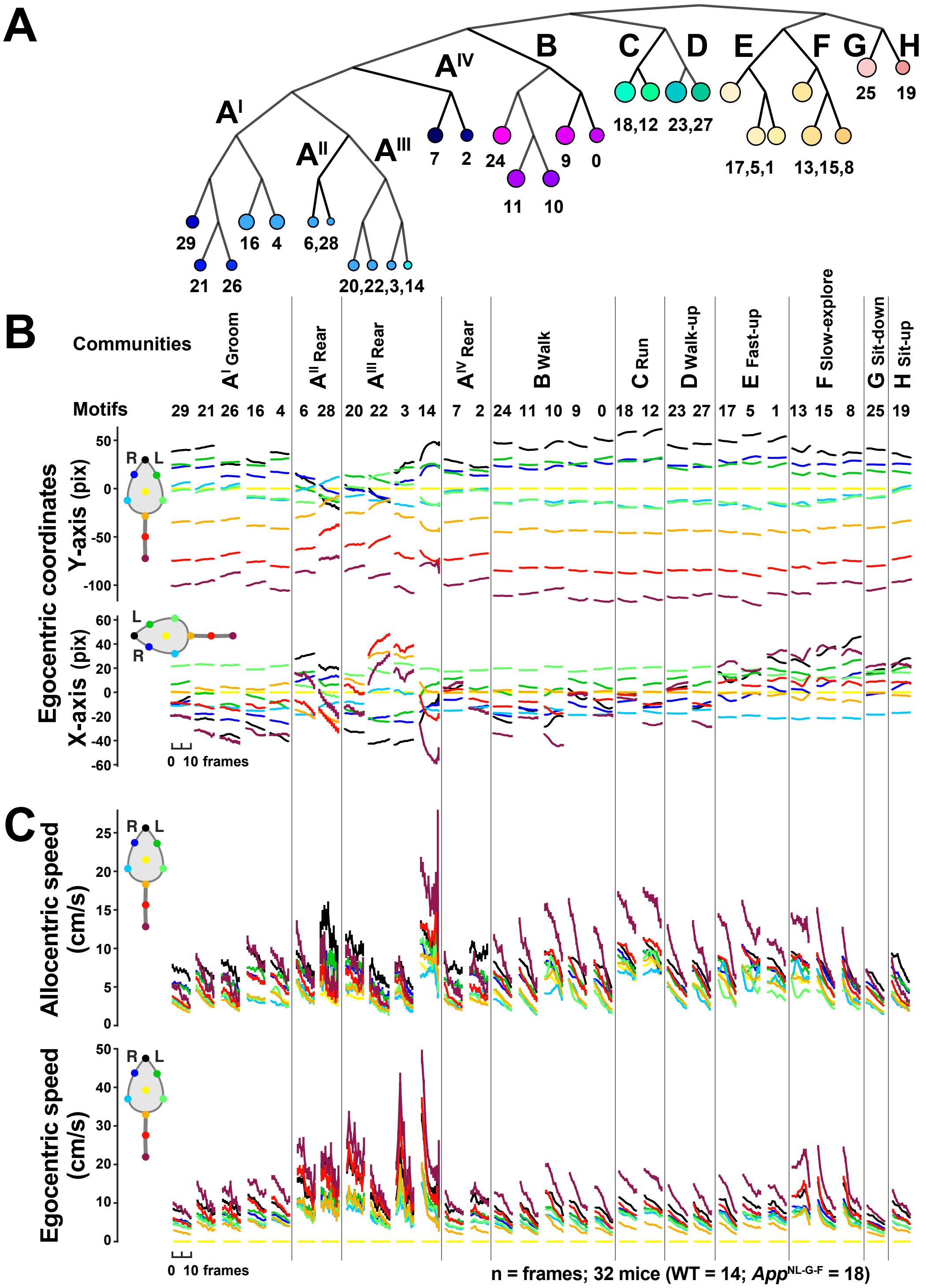

Figure S2, related to Figures 3 and 4. Hierarchical organization of motif kinematics in *App*$^{NL-G-F}$ mice.
(A) Hierarchical organization of motifs into communities as in Figure 4A. Motifs likely to occur consecutively were grouped together according to Equation 1.

(B) Kinematic analyses of the 30 motifs organized by community. Egocentric coordinates for the rostral–caudal (y-axis, top) and left–right (x-axis, bottom) axes of the nine body parts for the 30 identified motifs (n = 32; 18 *App*$^{NL-G-F}$ and 14 WT mice; 13 months of age). Points for each body part are the average location across each of the first 10 consecutive frames (or 400 ms) for every observation of a motif. Each data point is the mean and SEM of frame position for all 32 mice in the cohort.

(C) Allocentric (top, relative to arena) and egocentric (bottom, relative to mouse) speeds of the defined body parts across the first 10 consecutive frames (or 400 ms) of motif initiation in 13-month-old *App*$^{NL-G-F}$ mice (n = 18; 8 females and 10 males) and WT littermate controls (n = 14; 8 females and 6 males). Values are mean ± SEM of speed per frame for all 32 mice in the cohort.

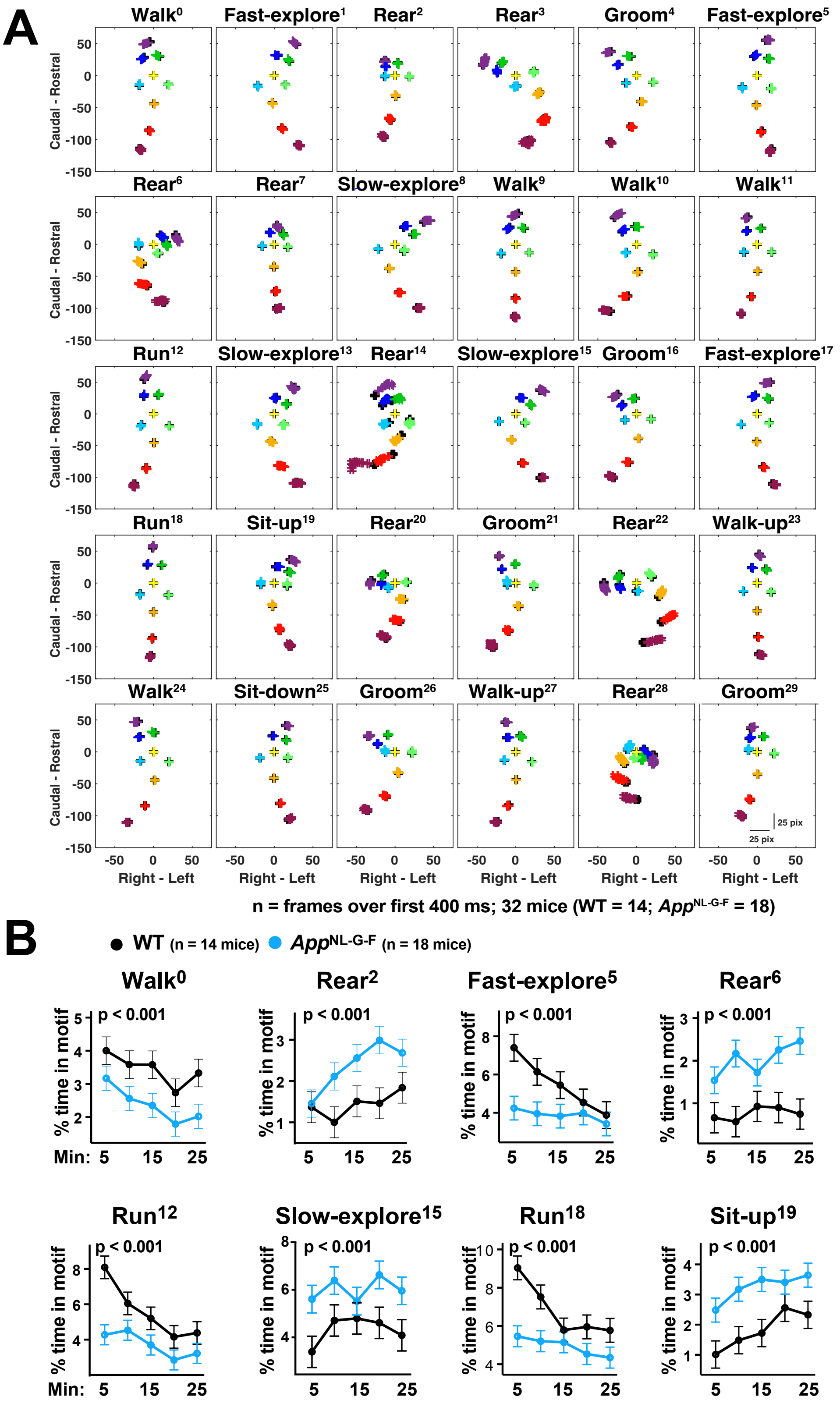

Figure S3, related to Figures 3 and 4. Positions of body parts for the 30 motifs and selected examples of motif usage differentially modulated by time identified in *App*$^{NL-G-F}$ mice.
(A) Motif-specific egocentric coordinates of the defined body parts for the 30 identified motifs across the first 10 consecutive frames (or 400 ms) from the motif initiation in 13-month-old *App*$^{NL-G-F}$ mice (n = 18; 8 females and 10 males) and WT littermate controls (n = 14; 8 females and 6 males). Each data point is the average position over observations (frames) for the full cohort (n = 32 mice) ± SEM. Error bars are generally smaller than the plus-symbol markers.

(B) Percentage of time spent in identified motifs during 25 minutes of exploration in an open circular arena. Motifs usages were strongly modulated by time (experience), and *App*$^{NL-G-F}$ mice exhibited impaired time-dependent responses. P values were determined by repeated one-way ANOVA. Values are mean ± SEM.

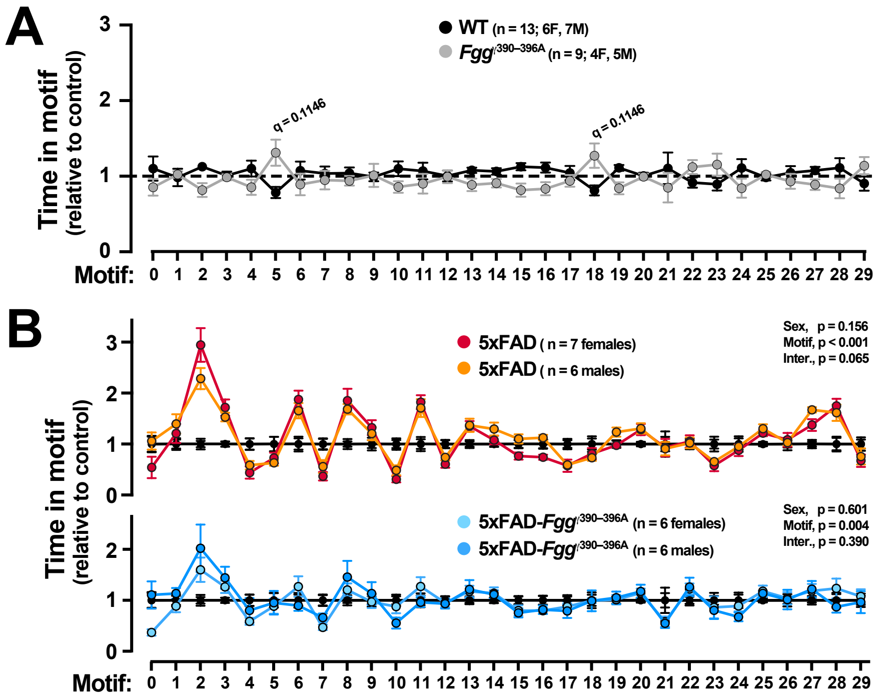

Figure S4, related to Figure 6. VAME motif use is consistent across sex and within the 5xFAD-*Fgg*$^{\gamma390\text{–}396A}$ control groups.

Spontaneous naturalistic behavior of 9-month-old 5xFAD mice (n = 13; 7 females and 6 males), 5xFAD-*Fgg*$^{\gamma390\text{–}396A}$ mice (n = 12; 6 females and 6 males), *Fgg*$^{\gamma390\text{–}396A}$ controls (n = 9; 4 females and 5 males), and NTG controls (n = 13; 6 females and 7 males) were recorded for 60 minutes in a circular open arena.

(A) Motif use (relative to a sex-matched *Fgg*$^{\gamma390\text{–}396A}$ and NTG combined control group) of 30 identified motifs for *Fgg*$^{\gamma390\text{–}396A}$ and NTG mice. Relative to the combined control group, *Fgg*$^{\gamma390\text{–}396A}$ mice and NTG mice displayed no significant differences in motif use. Significance was defined as $q < 0.05$ by false-discovery rate with Benjamini-Hochberg correction for multiple comparisons. Values are mean ± SEM.

(B) Motif use (relative to that of sex-matched controls) in female and male 5XFAD mice (top) and female and male 5xFAD-*Fgg*$^{\gamma390\text{–}396A}$ mice (bottom). Motif performance differed significantly across motifs, but motif performance within each genotype and usage trends across sexes were similar and no sex–motif interaction was observed, suggesting that 5xFAD and 5xFAD-*Fgg*$^{\gamma390\text{–}396A}$ genotypes affect males and females similarly. Sex and motif effects were determined by two-way ANOVA (p values). Values are mean ± SEM.

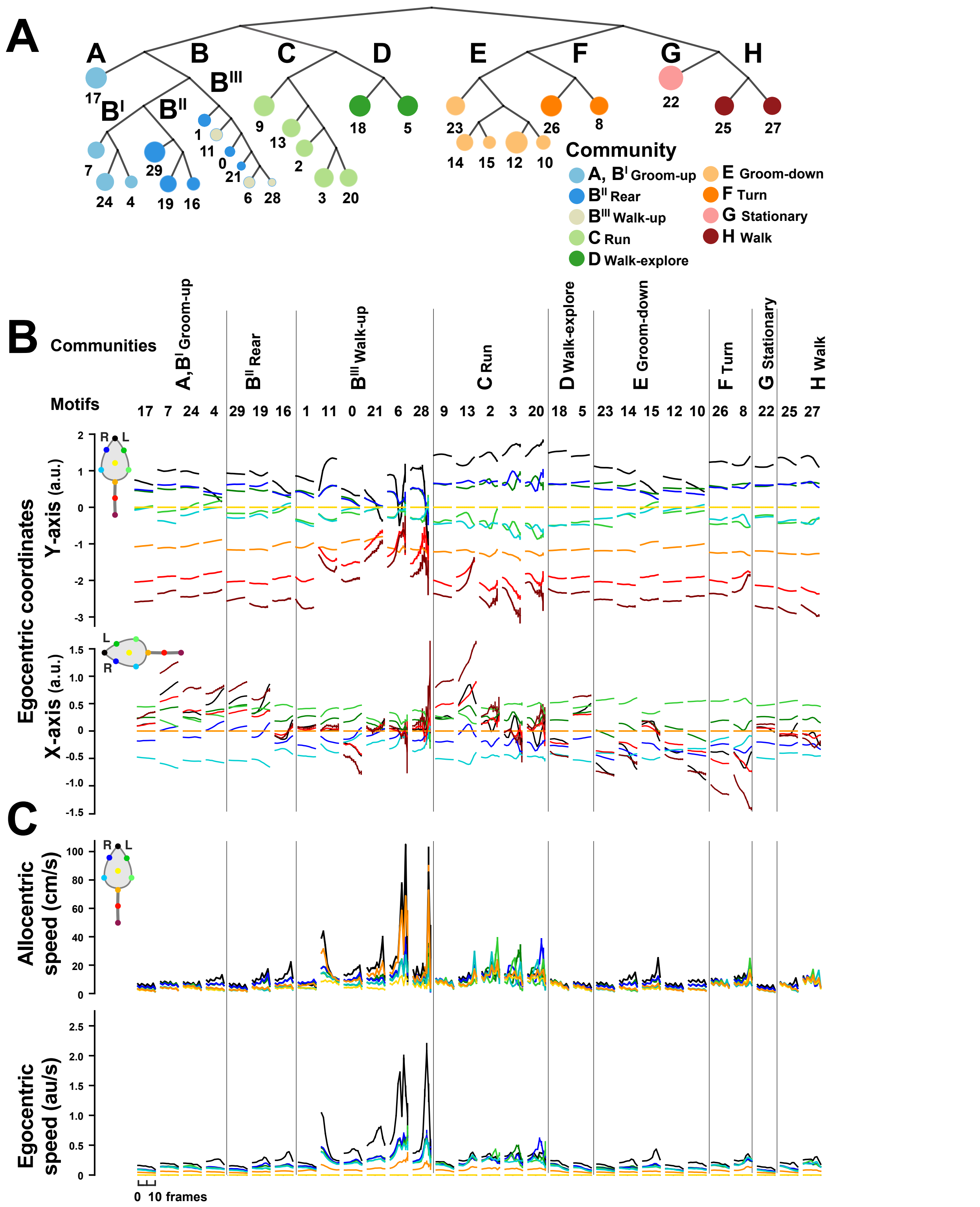

Figure S5, related to Figures 6 and 7. Hierarchical organization of motif kinematics in 5xFAD-*Fgg*$^{\gamma390-396A}$ mice.

(A) Hierarchical organization of motifs into communities as shown in Figure 7A for 5xFAD-*Fgg*$^{\gamma390-396A}$ mice. Motifs likely to occur consecutively were grouped together according to Equation 1.

(B) Kinematic analyses of the 30 motifs organized by community. Egocentric coordinates for the rostral–caudal (y-axis, top) and left–right (x-axis, bottom) axes of the nine body parts for the 30 identified motifs (n = 47; 13 5xFAD mice, 12 5xFAD-*Fgg*$^{\gamma390-396A}$ mice, 22 control mice; 9 months of age). Points for each body part are the average location across each of the first 10 consecutive frames (or 400 ms) for every observation of a motif. Each data point is the mean ± SEM of frame position for all 47 mice in the cohort.

(C) Allocentric (top, relative to arena) and egocentric (bottom, relative to mouse) speeds of the defined body parts across the first 10 consecutive frames (or 400 ms) of motif initiation. Mid-tail and tail tip points were omitted for ease of visualization. Values are mean ± SEM of speed per frame for all 47 mice in the cohort.

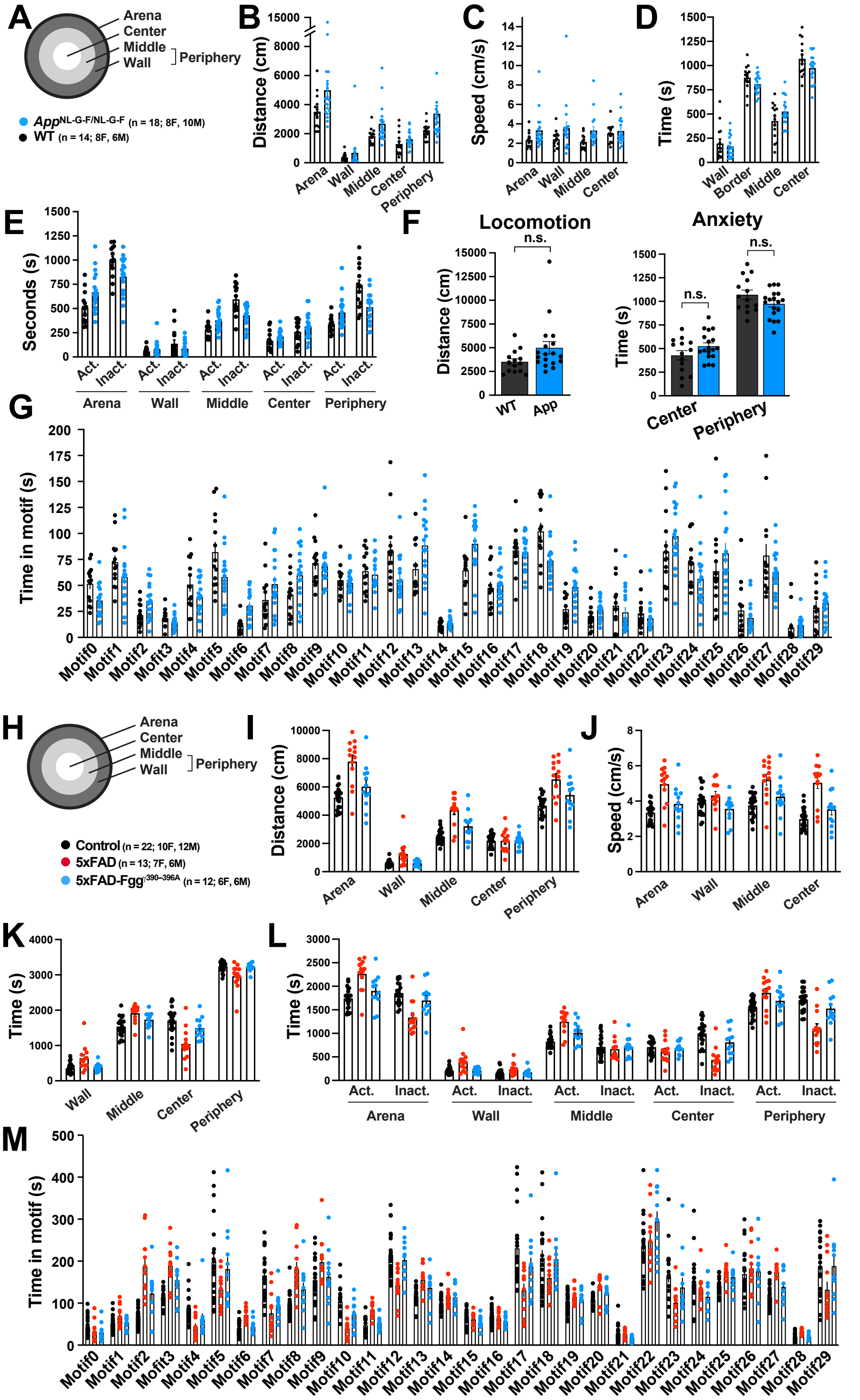

Figure S6, related to Figures 5 and 6. Machine learning and conventional behavioral open field outcomes for *App*$^{NL-G-F}$ and 5xFAD cohorts.

(A-G) 13-month-old *App*$^{NL-G-F}$ mice (n = 18; 8 females and 10 males) and WT littermate controls (n = 14; 8 females and 6 males) were tested in a circular open field arena for 25 min. Conventional outcomes were calculated for the total arena as well as for its center, middle, wall, and periphery (middle + wall) zones. Distance (B), speed (C), total time (D), and active and inactive time (E) were assessed in the arena (total), center, middle, wall, and periphery. The classifier analyses included all 23 variables.

(F) Conventional behavior readouts assessing locomotor function (total distance travelled) and anxiety (time in center vs. periphery) showed no significant differences between genotypes. $p > 0.05$ by Student's t-test. Values are mean ± SEM

(G) Motif use in *App*$^{NL-G-F}$ and WT mice. The classifier analyses included all 30 variables.

(H-M) 8–10-month-old 5xFAD mice (n = 13; 7 females and 6 males), 5xFAD-*Fggγ*$^{390-396A}$ mice (n = 12; 6 females and 6 males), and controls (n = 22; 10 females and 12 males) were tested in a circular open field arena for 60 min. Distance (I), speed (J), total time (K), and active and inactive time (L) were assessed in the arena (total), center, middle, wall, and periphery. The classifier analyses included all 23 variables.

(M) Motif use in 5xFAD, 5xFAD-*Fggγ*$^{390-396A}$, and control mice. The classifier analyses included all 30 variables.

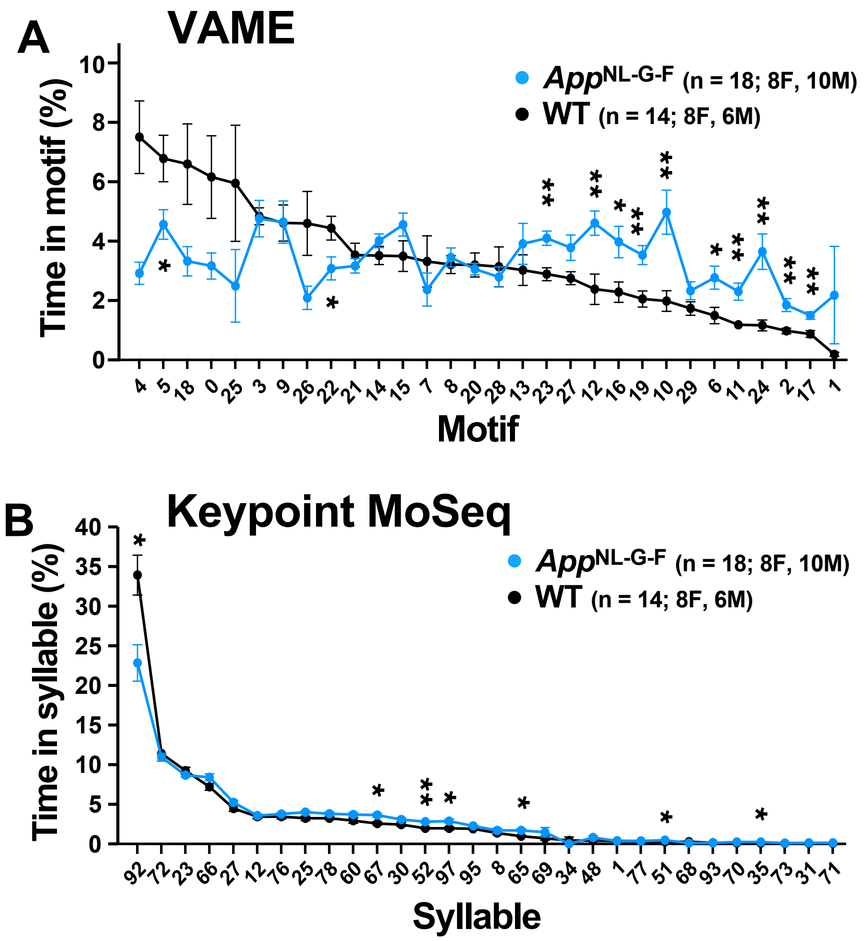

Figure S7, related to Figure 3. Comparison of unsupervised behavioral segmentation approaches.
(A and B) Side-by-side comparisons of VAME (A) and keypoint-MoSeq (B) segmentation results obtained from the same videos for WT and *App*$^{NL-G-F}$ mice. Values are percentage time in 30 motifs (A) or the 30 most-used syllables (B). **q < 0.01, *q < 0.05 by FDR-BH. Values are mean ± SEM.

Supplemental Table 1, related to Figure 5. Leave-one-out log-likelihood estimate comparisons for orders 0 through 3 for the Markov chain model of community transitions in *App*$^{NL-G-F}$ mice. Using the log pseudo Bayes factor as a metric of model comparison, the Markov models of orders 1 and 0 provide better fits to the data for both WT and *App*$^{NL-G-F}$ mice than models of orders 2 and 3.

**Order**	**1 vs 0**	**2 vs 0**	**3 vs 0**	**2 vs 1**	**3 vs 1**	**3 vs 2**
**WT (all mice)**	21474.02	-28124.31	-379255.44	-49598.33	-400729.46	-351131.13
**WT (females)**	13036.96	-15926.07	-229885.45	-28963.04	-242922.41	-213959.37
**WT (males)**	8437.06	-12198.24	-149369.99	-20635.30	-157807.05	-137171.75
***App* (all mice)**	23498.40	-22962.84	-495831.01	-46461.24	-519329.40	-472868.16
***App* (females)**	8995.67	-20513.74	-310021.62	-29509.41	-319017.29	-289507.88
***App* (males)**	14502.73	-2449.10	-185809.39	-16951.83	-200312.12	-183360.28
